# Versatile Chemo-Biocatalytic Cascade Driven by a Thermophilic and Irreversible C–C Bond-Forming α-Oxoamine Synthase

**DOI:** 10.1021/acssuschemeng.3c00243

**Published:** 2023-05-16

**Authors:** Ben Ashley, Arnaud Baslé, Mariyah Sajjad, Ahmed el Ashram, Panayiota Kelis, Jon Marles-Wright, Dominic J. Campopiano

**Affiliations:** † School of Chemistry, University of Edinburgh, Joseph Black Building, David Brewster Road, Edinburgh EH9 3FJ, United Kingdom; ‡ Biosciences Institute, Faculty of Medical Sciences, Newcastle University, Newcastle upon Tyne NE2 4HH, United Kingdom

**Keywords:** *Th*AOS, α-aminoketone, Knorr pyrrole reaction, biocatalyst, α-oxoamine synthase

## Abstract

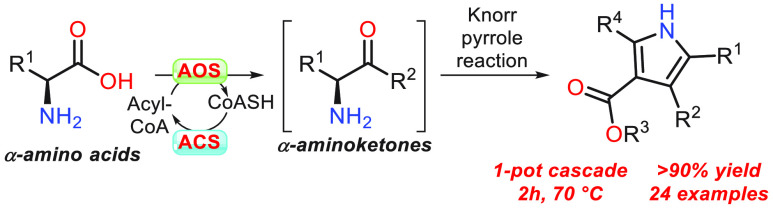

We report a chemo-biocatalytic cascade for the synthesis of substituted pyrroles, driven by the action of an irreversible, thermostable, pyridoxal 5′-phosphate (PLP)-dependent, C–C bond-forming biocatalyst (*Th*AOS). The *Th*AOS catalyzes the Claisen-like condensation between various amino acids and acyl-CoA substrates to generate a range of α-aminoketones. These products are reacted with β-keto esters in an irreversible Knorr pyrrole reaction. The determination of the 1.6 Å resolution crystal structure of the PLP-bound form of *Th*AOS lays the foundation for future engineering and directed evolution. This report establishes the AOS family as useful and versatile C–C bond-forming biocatalysts.

## Abstract 2

We describe a fast method that combines chemical and biological reactions that allows the preparation of a series of organic molecules using simple, easily recycled starting materials.

α-Aminoketones are prominent in the biosynthesis of a variety of important natural products. They are also a versatile and highly functionalizable motif in organic chemistry and are gaining increasing attention as valuable starting materials and intermediates for synthesis.^38−6^ Due to the difficulty of obtaining unprotected α-aminoketones synthetically, several biocatalytic routes toward this key synthetic building block have been explored.^39^ One method applied the popular pyridoxal 5′-phosphate (PLP)-dependent transaminases (TAs) to transfer an amino group from an amine donor to a diketone acceptor.^5^ The resulting α-aminoketone was transformed *in situ* into pyrazines by oxidative dimerization, as well as pyrroles using a Knorr pyrrole reaction (KPR).^15^ A similar TA-mediated amine borrowing strategy, coupled with a KPR, was recently used to generate a small library of pyrroles.^7^ TAs are useful biocatalysts but suffer from the problem of reversibility of the amine transfer step.^8^

An alternative route to the α-aminoketone building block would be to use a different PLP-dependent biocatalyst that does not rely on reversible amine transfer. α-Oxoamine synthases (AOS) fall within this category. These enzymes catalyze the Claisen-like, decarboxylative condensation of an α-amino-acid (AA) with an acyl-CoA-thioester (Figure 1A; Figure S1). This irreversible biocatalytic reaction generates α-aminoketones with release of CoASH and CO_2_ (Scheme 1A). The structure and mechanism of members of the AOS family have been studied for a number of years since they play essential roles in the biosynthesis of important metabolites including heme, biotin, sphingolipids, amino acids and polyketides.^11−4^ Their narrow substrate specificity, moderate stability, and the requirement for expensive acyl-CoA thioester substrates have so far precluded the exploitation of AOS enzymes as synthetically useful biocatalysts. However, recent studies indicate that they have potential as stand-alone biocatalysts in the preparation of deuterated drug targets, as well as combined with other enzymes in a two-step cascade for the synthesis of α-aminoketones.^40,1^

**Scheme 1:**
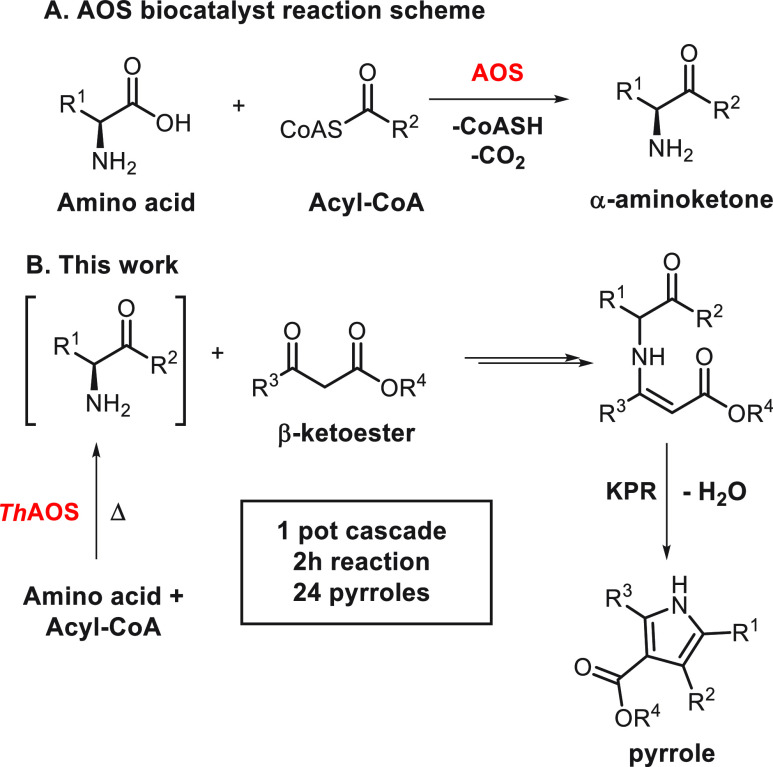
Overview of Chemo-Biocatalytic Cascade. (A) α-Oxoamine Synthases (AOSs) Catalyse the Claisen-Like Condensation of Amino Acids and acyl-CoAs to Generate Chiral α-Aminoketones. (B) α-Aminoketones Generated by *Th*AOS React with β-Ketoesters (BKEs) *In Situ* to Produce Substituted Pyrroles via a Knorr Pyrrole Reaction (KPR)

**Figure 1:**
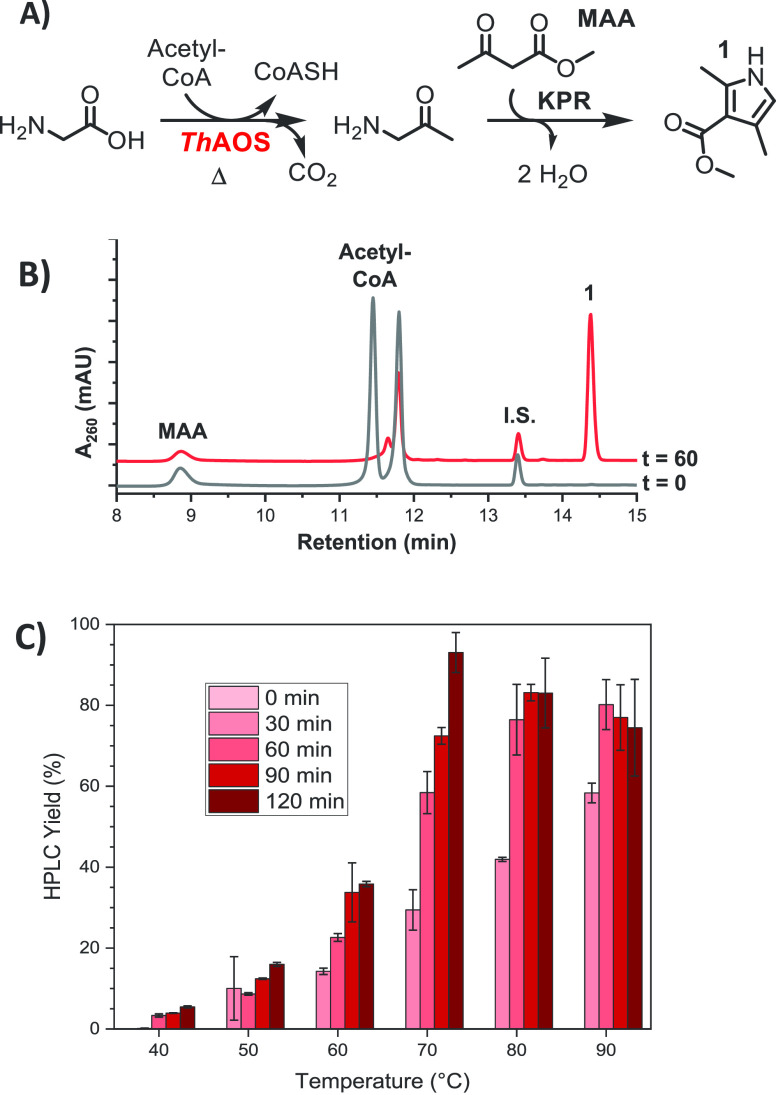
The AOS/KPR chemo-biocatalytic cascade. (A) The AOS-KPR cascade toward pyrrole **1**. (B) HPLC trace of the reaction at *t* = 0 min (black line) and *t* = 60 min (red line). The internal standard (I.S.) was benzoate. (C) Time and temperature optimization of the *Th*AOS/KPR coupled cascade.

For a useful hypothetical AOS biocatalyst, three main properties would be desirable: high activity; thermostability; and, most importantly, a broad substrate scope. This would permit application of the AOS as a C–C bond-forming biocatalyst in as broad an array of conditions and syntheses as possible. Such an enzyme, *Th*AOS from *Thermus thermophilus*, has been previously isolated.^14^ Herein, we show that *Th*AOS can be employed as a robust C–C bond-forming biocatalyst for the formation of a broad range of α-aminoketones. As an example of its utility, we couple the *Th*AOS-catalyzed condensation reaction *in situ* with a chemical step, the KPR, to generate various substituted pyrroles (Scheme 1B) in good yields and short reaction times under mild conditions.

Recombinant *Th*AOS (Uniprot: Q5SHZ8, purchased as codon-optimized clone from GenScript) was first expressed in *E. coli* BL21 (DE3) and purified using standard chromatographic methods, yielding >70 mg enzyme per liter of culture. Purified *Th*AOS was yellow and displayed a characteristic absorbance spectrum of PLP-binding enzymes (Figures S2A, B; S3A, B). Due to the release of CoASH, the catalytic activity of AOS enzymes can be monitored using the thiol detection reagent 5,5′-dithio-bis(2-nitrobenzoic acid), (DTNB, Figure S3C).^17^ This coupled, colorimetric assay was used to screen *Th*AOS for activity against a panel of AAs and acyl-CoA thioesters at 50 °C. *Th*AOS was active with AAs (*S*)-2-aminobutyric acid (l-Aba), l-Ala, Gly and l-Ser, and acetyl-, propionyl-, butyryl-, hexanoyl-, and octanoyl-CoA (Figure S3D, E; Tables S1–S3). The 20 reactions reported by this screen were later verified by mass spectrometry. This substrate scope sets *Th*AOS apart from other wild-type AOS enzymes, which are typically highly substrate specific, especially for the AA susbtrate.^18^ Two recently published AOS biocatalysts, SxtA and Alb29, catalyzed 9 and 7 reactions, respectively, but only with one AA each (l-Arg for SxtA and l-Glu for Alb29).^4,1^ A more recent AOS biocatalyst, the fusion enzyme BioWF from *Corynebacterium amycolatum*, was reported to catalyze 12 unique reactions, mostly with l-Ala but also including two reactions with Gly and l-Ser.^19^ Therefore, it appears that *Th*AOS is unique in terms of the diversity of substrate acceptance for both AA and acyl-CoA thioester substrates. We next characterized *Th*AOS thermostability, finding it stable at temperatures of up to 80 °C over 3 h (Figure S4).

Having characterized the scope of *Th*AOS as a C–C bond-forming biocatalyst, we next turned to coupling the *Th*AOS biocatalytic step with the KPR *in situ*. As a model reaction, we targeted pyrrole **1**, which would be derived from Gly and acetyl-CoA as the *Th*AOS substrates, coupled with methyl acetoacetate (MAA, Figure 1A). Pyrrole **1** was prepared chemically from aminoacetone hydrochloride and MAA and used as a standard to develop a quantitative HPLC assay (Figure 1B). We first optimized the KPR under buffered aqueous reaction conditions to maximize its compatibility with the *Th*AOS reaction. An organic cosolvent was required to solubilize MAA. Solvents were screened, and acetonitrile was found to be the best (Figure S5A). Furthermore, pH 7.6 was found to be optimal (Figure S5B).

With optimal KPR conditions in hand, a *Th*AOS-driven chemo-biocatalytic cascade toward pyrrole **1** was then established. An excess of the starting materials Gly and MAA (32 mM) were used, with acetyl-CoA (2 mM) as the limiting reagent in the presence of *Th*AOS (1 mg mL^–1^, 22 μM). Reactions were performed at 10 °C intervals at 40–90 °C and monitored by HPLC (Figure 1C). Good yields of 1 (>90%, TTN_*Th*AOS_^app^ = 79) were obtained after 2 h at 70 °C. Surprisingly, the reaction at 90 °C, beyond the stability limit of *Th*AOS, also gave a good yield (80%), after 1 h. The irreversibility of the PLP-dependent, decarboxylative reaction is advantageous to this cascade in comparison to PLP-dependent, TA-based methods which require high concentrations of an amino donor to overcome the issues of reversible equilibration.^5^ The thermal stability of *Th*AOS relative to mesophilic TA biocatalysts in previous studies also permitted heating of the reaction mixture, facilitating acceleration of the KPR chemical step which would not otherwise be possible.^5^

Interestingly, the final yield of **1** was found to be dependent both on [Gly]_0_ and [MAA]_0_ (Figure S5C, D), with reductions in the initial concentrations of either of these reagents reducing the final yield of the pyrrole after 2 h under the otherwise similar conditions. Upon further optimization, *Th*AOS loading was reduced to 0.1 mg mL^–1^ (2.2 μM) without concomitant loss of yield, improving TTN_ThAOS_^app^ to 810 (Figure S6).

We next looked to demonstrate the broad scope of our chemo-biocatalytic cascade. When the 20 previously identified *Th*AOS-driven condensations were carried out in the presence of MAA under optimized conditions, the formation of the corresponding pyrrole products (**1**–**20**) was confirmed by LC-ESI-MS (Figures S7–S11). Signals for each of the 20 pyrroles were observed, and in some cases, the α-aminoketone intermediates were also visible (**14b**, **15b**, **17b**–**20b**). This demonstrates the broad utility of *Th*AOS as a biocatalyst in the context of a chemo-biocatalytic cascade.

We next probed the tolerance of the cascade for alternative acceptor substrates to MAA in the KPR (**21a**–**28a**, Table 1), including β-ketoketones (BKKs) as well as β-ketoesters (BKEs). Pyrrole standards (**1**, **5**, **21**, **25**, **26**, **28**) were obtained (see S.I.) and assayed by HPLC (Figure S12). We selected a diverse range of BKKs and BKEs (Table 1). Upon incubation of the KPR reagents with *Th*AOS, Gly and acyl-CoA under the optimized conditions, we observed the formation of five new pyrrole products (**5**, **21**, **25**, **26** and **28**) by comparison with HPLC synthetic standards, demonstrating the cascade to be compatible with a variety of KPR acceptor reagents. No products were observed under any conditions when R^4^ was adjusted to bulkier substituents than Et (**22a**–**24a**), but otherwise, HPLC yields ranged from 54% (**26**) to 93% (**1**). Although pyrrole **21** was only produced in 28% yield over 2 h, this was improved to 47% by extending the reaction time to 4 h. This implies that the loss of conversion is due to a reduction in the rate of the KPR and influenced by the nature of the substituent at R.^4^

**Table 1:**
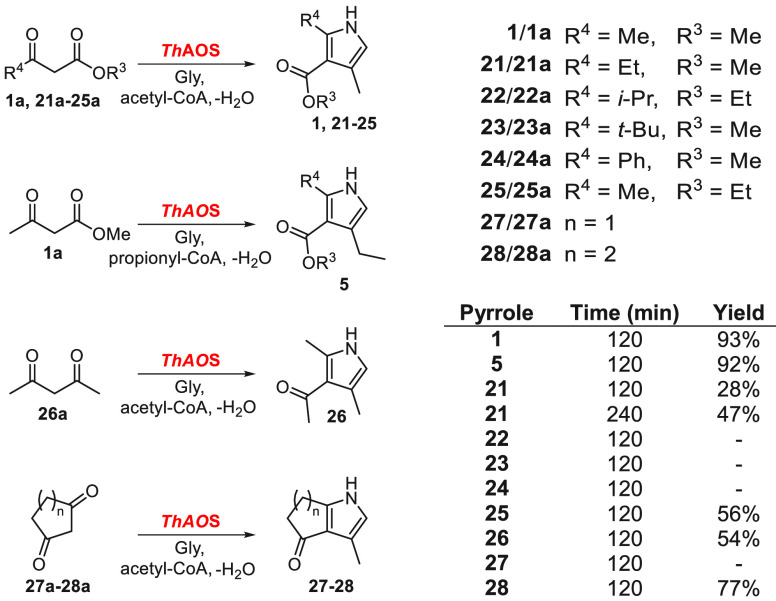
Formation of Substituted Pyrrole Products Using the ThAOS Biocatalyst with the Alternative Knorr Pyrrole Reaction Acceptor Substrates^a^

a Reactions were performed by incubation of Gly (32 mM) with *Th*AOS (1 mg mL^–1^), acyl-CoA (2 mM) and BKE/BKK (32 mM) in aqueous buffer (100 mM HEPES, 150 mM NaCl, pH 7.5) at 70 °C, and % yields determined by HPLC.

To show that our coupled chemo-biocatalytic system is synthetically useful, we next employed our cascade system to prepare pyrrole **1** at milligram scale from Gly, MAA, and acetyl-CoA. After reaction completion and workup, we isolated 16.4 mg of **1** from 100 mg of acetyl-CoA (87% yield).

While attractive in other aspects, the *Th*AOS/KPR cascade for pyrrole synthesis consumes stoichiometric quantities of acyl-CoAs over the course of the reaction (Figure 1). As with other enzyme cofactors, CoASH and its acyl-thioester derivatives are expensive. Therefore, it would be more economical if the acyl-CoA thioester substrate could be generated and regenerated *in situ* by recycling the CoASH byproduct (Figure 2A).^20,21^ This could be achieved by including an auxiliary biocatalyst, such as an acetyl-CoA synthetase (ACS), which ligates CoASH to a carboxylic acid with consumption of adenosine triphosphate (ATP) and release of adenosine monophosphate (AMP) and pyrophosphate (PPi).

**Figure 2:**
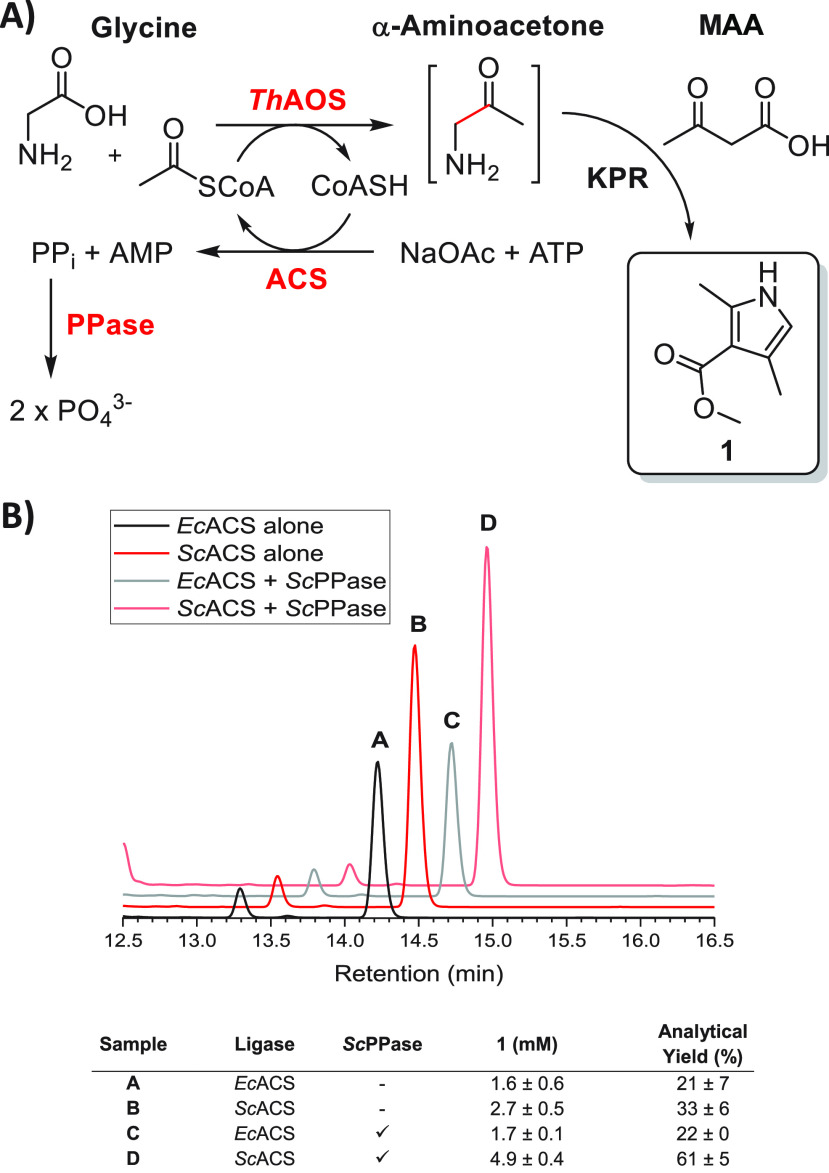
Formation of pyrrole **1** via a CoA-regenerating chemo-biocatalytic cascade. (A) Generation of pyrrole **1** by coupling a KPR with three biocatalysts (*Th*AOS, ACS, and PPase). Acetyl-CoA is generated *in situ* by the ATP-dependent, ACS-catalyzed condensation of sodium acetate and CoASH. The inhibitory pyrophosphate (PP_i_) byproduct is hydrolyzed by PPase. The *Th*AOS condenses glycine and acetyl-CoA to generate the α-aminoacetone, which couples to methyl-acetoacetate by KPR to give pyrrole **1**. (B) Formation of pyrrole **1** using either *Ec*ACS (sample A) or *Sc*ACS (sample B). The addition of a PPase led to no change in the amount of pyrrole **1** (sample C) but a 1.8 tmes increase in the formation of **1** when the *Sc*ACS and *Sc*PPase are combined (sample D). Detailed conditions are found in the S.I.

To test this, we used commercially available *Saccharomyces cerevisiae* ACS (*Sc*ACS, CAS: 9012-31-1), as well as a recombinant ACS cloned and expressed from *Escherichia coli* (*Ec*ACS, Uniprot: P27550, Figure S2C) in a cascade toward pyrrole **1**. The acetyl-CoA starting material included in previous reactions was omitted and replaced with acetate (32 mM), CoASH (1 mM), limiting reagent ATP (8 mM), reducing agent tris(2-carboxyethyl)phosphine (TCEP, 1 mM), and an ACS biocatalyst (*Ec*ACS or *Sc*ACS, 1 mg mL^–1^, ∼14 μM) in order to generate acetyl-CoA *in situ*. The concentrations of all other reagents were kept the same.

When the reactions were performed overnight at 37 °C and analyzed by HPLC, pyrrole **1** was observed only in the presence of all reaction components. *Sc*ACS was the superior cofactor-recycling biocatalyst, giving a 33% yield from ATP (2.67 mM **1**, Figure 2B, samples A and B). Furthermore, when a pyrophosphatase (*S. cerevisiae* PPase, CAS: 902-82-2) was included to counter any potential product inhibition of *Sc*ACS by PP_i_,^22,23^ the yield of **1** from the *Sc*ACS-catalyzed cascade improved from 33% to 61% (4.88 mM **1**, Figure 2B). This represents a significant increase relative to the previous acetyl-CoA-based reactions, which were limited to 2 mM. In terms of moles of product generated per mole of CoASH consumed, they also increased from 0.9−0.93, to 4.88 a >5-fold improvement. We therefore present the successful establishment of a three-enzyme chemo-biocatalytic cascade, starting from simple starting materials Gly, acetate, and ATP, that together generate substituted pyrrole target molecules with *Th*AOS as the key C–C bond-forming biocatalyst.

The successful application of *Th*AOS for pyrrole synthesis inspired us to understand the molecular basis for the broad substrate specificity displayed by this enzyme. We determined the X-ray crystal structure of PLP-bound *Th*AOS at a 1.6 Å resolution in three different space groups (Figure 3; Figures S19 andS20; Table S4, PDB accession codes: 7POA, 7POB, and 7POC). The phase problem was solved by molecular replacement using the structure of *Coxiella burnetii* KBL (PDB: 3TQX, Figure S21).^24^

**Figure 3:**
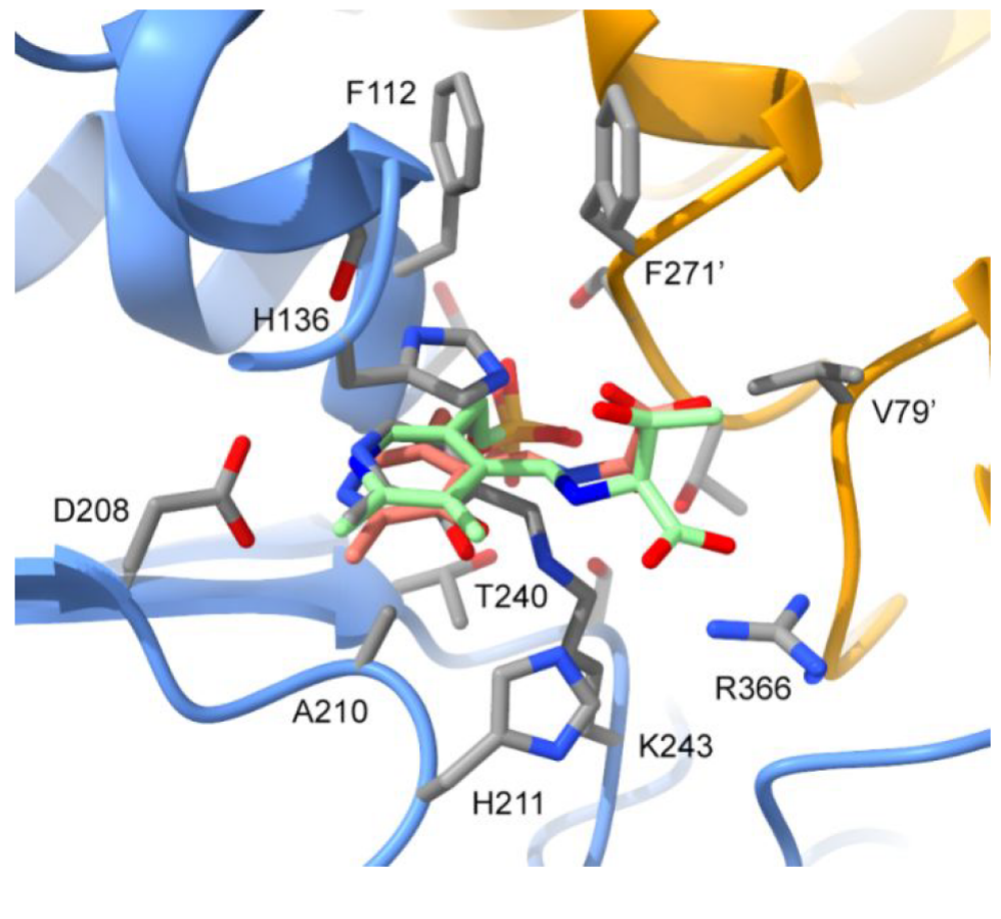
Structural insights of ThAOS substrate binding. The active site of *Th*AOS, showing the PLP cofactor bound to K243. The *Th*AOS structure (PDB: 7POA) was aligned with the PLP-l-Thr PLP external aldimine complex of *Cupriavidus necator* KBL (PDB: 7BXQ, green)^2^ and the *S. paucimobilis* SPT PLP-l-Ser external aldimine complex (PDB: 2W8J, pink).^17^

Like other members of the AOS family, *Th*AOS forms a tight homodimer with the PLP cofactor bound at the interface between the two monomers (Figure 3; Figures S19, S20), typical of type IV PLP-binding enzymes.^25^ The active site is highly similar to other AOS family members (Figure 3; Table S4).^4,26−30^ When the structure of the PLP-bound *Th*AOS was compared with the PLP-l-Thr external aldimine complex of the *C. necator* KBL (PDB: 7BXQ),^2^ it revealed that the carboxylate group of l-Thr is proposed to sit close to the conserved *Th*AOS Arg366 in an apparent salt-bridge interaction. Similarly, in the *S. paucimobilis* SPT-l-Ser complex (PDB: 2WJ8), two arginine residues (Arg370 and Arg390) play key roles in substrate binding and catalysis.^17^ We predict that Arg366 is involved in similar functions in *Th*AOS, allowing the biocatalyst to accept the observed broad range of AAs. The acceptance of acyl-CoA substrates ranging from C_2_–C_8_ is more difficult to understand since there is a lack of high-resolution structures of bacterial AOS:acyl-CoA complexes. However, the observation that *Th*AOS can use various acyl-CoAs suggests that the binding site is suitably dynamic to accommodate a broad range of acyl chains. Furthermore, a sequence alignment of *Th*AOS with 7 other AOS members highlights conserved residues that are potentially involved in substrate binding and catalysis (Figure S21). These will be ideal candidates for future engineering.

In summary, we have shown that an unusual thermophilic AOS biocatalyst (*Th*AOS) displays an inherently broad substrate scope that can be used to efficiently generate α-aminoketones at elevated temperatures. Our study is the first to make use of the full AOS catalytic cycle to generate a range of >20 α-aminoketone derivatives. This biocatalytic step was coupled with a panel of acceptor substrates in a compatible KPR to generate a library of 24 pyrrole products. We further showed that one of the key obstacles in the application of AOS biocatalysts, the use of the acyl-CoA thioester substrate, can be overcome via biocatalytic recycling of the CoASH factor. The issue of PP_i_ inhibition of the ACS biocatalyst was successfully tackled via inclusion of a hydrolytic PPase. Further optimization of these auxiliary biocatalysts should lead to improved yields of the desired target molecules. The final key result was the determination of the 1.6 Å resolution crystal structure of *Th*AOS with the bound PLP cofactor. This molecular insight paves the way for future engineering of *Th*AOS to expand its substrate range—an endeavor which will be aided by the inherent and unique thermostability of *Th*AOS. This should permit the inclusion of beneficial mutations without sacrificing the catalytic activity and stability of the overall fold.

Biocatalysis is a rapidly expanding area that is making key contributions in sustainable synthetic chemistry, chemical manufacturing, and the preparation of clinically used drugs.^31−33^ The field benefits from a useful and comprehensive database of enzymes from which to select and screen for the desired chemical transformation.^34^ RetroBioCat provides a collection of tools for biocatalytic cascade design, working backward from the target molecule in a retrosynthetic manner.^35^ The PLP-dependent TAs are proven biocatalysts for amine synthesis, with many examples to choose from in the inventory. We hope that the results using *Th*AOS, combined with the recently published studies on other AOSs, will encourage the addition of members of this versatile family to this growing biocatalyst database. In future work, we also suggest that directed evolution/engineering of *Th*AOS, facilitated by its inherent thermostability, and further improvements in acyl-CoA regeneration, will expand the synthetic utility of AOS enzymes.^36,37^ These could be used as stand-alone biocatalysts or be incorporated in multistep, chemo- and/or biocatalytic cascades.

## Supporting Information Available

Details of enzyme purification, assay, kinetics, product formation, mass spectrometry, NMR, and structure determination (PDF)

## Accession Codes

*Th*AOS, Uniprot sequence: Q5SHZ8. *Ec*ACS, Uniprot sequence: P27550. PDB codes: 7POA, 7POB, 7POC.
