# Supplementary material for: Versatile Chemo-Biocatalytic Cascade Driven by a Thermophilic and Irreversible C–C Bond-Forming α-Oxoamine Synthase
Source: ACS Sustain Chem Eng. 2023 May 16;11(21):7997–8002. doi: 10.1021/acssuschemeng.3c00243 (PMC10230504; doi:10.1021/acssuschemeng.3c00243)
Supplement: Supplementary file 1 — sc3c00243_si_001.pdf [file sc3c00243_si_001.pdf]

# Supporting Information for

## "A Versatile Chemo-Biocatalytic Cascade Driven by a Thermophilic and Irreversible C-C Bond-Forming α-Oxoamine Synthase”.

Ben Ashley,[a] Arnaud Baslé,[b] Mariyah Sajjad,[a] Ahmed el Ashram,[a] Panayiota Kelis,[a] Jon Marles-Wright[b] and Dominic J. Campopiano[a]*

[a]School of Chemistry, University of Edinburgh, David Brewster Road, Edinburgh, EH9 3FJ, UK
[b]Biosciences Institute, Faculty of Medical Sciences, Newcastle University, Newcastle upon Tyne, NE2 4HH, UK

Corresponding author: *Dominic.Campopiano@ed.ac.uk

# Contents


1. General Methods & Materials ....3
2. Enzyme Expression and Purification ....3
3. Assays ....3
4. Preparation of Pyrrole Standards (**1, 5, 21, 25, 26, 28**) ....6
5. Scaled-Up Chemo-Enzymatic Synthesis of Pyrrole (**1)** ....9
6. Formation of Pyrroles by the coupled *Th*AOS/KPR cascade ....9
7. X-Ray Data Collection and Model Refinement ....10
8. Mechanism of AOS Enzymes ....11
9. Purification Recombinant Biocatalysts (*Th*AOS and *Ec*ACS) ....12
10. Assay of Recombinant *Th*AOS ....13
11. Substrate Scope *of Th*AOS ....14
12. Thermal stability of *Th*AOS ....17
13. Optimisation of the Formation of Pyrrole **1** by the chemo-biocatalytic *Th*AOS-KPR cascade. ..18
14. Detection of Pyrroles Products (**1-20**) by EIC LC ESI-MS. ....20
15. Analysis of Pyrroles (**1, 5, 21, 25, 26** and **28**) by HPLC. ....23
16. $^{1}H$ and $^{13}C$ NMR Spectra for Pyrroles (**1, 5, 21, 25, 26, 28**). ....25
17. X-Ray Structure of the PLP-Bound Form of *Th*AOS. ....31
18. Sequence and Structural Alignments ....33
19. Crystallographic Data ....34
20. DNA and Protein Sequences ....35
21. References ....37

# 1. General Methods & Materials

**Methods**
NMR spectra were recorded on a Bruker Avance III 500 MHz or a Bruker CryoProbe Prodigy 500 MHz, and the solvent was $CDCl_3$.

**Materials**
Commercially available standards, solvents and reagents were purchased from Avanti Lipids, Fluorochem, Sigma Aldrich, Cambridge BioScience and Thermo Fisher Scientific and were used without any further purification.

# 2. Enzyme Expression and Purification

**Expression of *Th*AOS**
A single colony of *E. coli* BL21 (DE3) cells containing a pET28a-based plasmid encoding *Th*AOS with a TEV-cleavable N-terminal $His_6$ tag was used to inoculate a 5 mL overnight culture of LB containing 30 ug/mL kanamycin. After overnight shaking at 37 °C the culture was used to inoculate larger cultures of 1L LB media in 2L Erlenmeyer flasks, which were then grown with shaking at 180 rpm at 37 °C until the $OD_{500}$ was 0.6-0.8. The culture was then induced with 0.25 mM IPTG overnight at 16 °C. Cells were harvested by centrifugation and cell pellets were stored at -20 °C.

**Purification of *Th*AOS**
*Th*AOS cell pellets (~10 g) were resuspended in HEPES buffer (20 mM, 150 mM NaCl, 5% glycerol, pH 7.5) in the presence of PLP (25 µM) before 30s on/off sonication for 15 minutes. Cell debris was then pelleted by centrifugation at 10,000xg for 45 minutes and the supernatant was filtered using a 0.45 µm filter. The clarified cell lysate was loaded onto a pre-equilibrate GE Healthcare Ni affinity column, washed with HEPES buffer to remove non-binding proteins and eluted with a gradient of 0-500 mM imidazole with fractionation. Yellow fractions were the concentrated and loaded onto a pre-equilibrated Superdex S200 column before elution with 120 mL HEPES buffer. The enzyme eluted at 65-80 mL, yielding highly pure enzyme. Enzyme concentration was determined by Bradford assay and was then concentrated to 20 mg/mL stocks and stored at -80 °C. Purity was assessed using standard SDS-PAGE electrophoresis.

**Purification of *Ec*ACS**
The purification EcACS used essentially the same method as for ThAOS.

# 3. Assays

**UV-Vis Spectroscopy**
UV-vis measurements were performed using a Varian Cary UV-Vis spectrophotometer. Spectra were baselined against HEPES buffer and enzyme was diluted until $A_{420}$ was approximately 0.3 AU. Amino-acid substrates were titrated in from 100 mM stocks in HEPES buffer and mixed before recording of

the new spectrum (amino-acid binding was found to be rapid, precluding the need for any incubation period). Spectra were normalised to account for the increasing volume of the sample due to the titration. In the case of L-Cys, 1 mM substrate was added from a 100 mM stock and incubated for 24 hours, and spectra were recorded at 60 minute intervals.

**$K_d$ Calculations**

To determine the $K_d$ for a given substrate spectral changes at 431 nm were recorded as a function of substrate concentration and plotted.

These plots were then fitted according to the equation below.

$$\Delta A_{obs} = \frac{\Delta A_{max}[L - amino\ acid]}{\mathrm{K}_d + [L - amino\ acid]}$$

This procedure was performed with pure *Th*AOS and the results are summarised in table S2.

**Colorimetric DTNB Activity Assay**

Measurements were performed in a BioTek Synergy HT 96-well plate reader. In all cases *Th*AOS was added last, after the acyl-CoA substrate had been incubated with the DTNB mixture for 10+ minutes to allow contaminating CoASH to react off. Measurements were performed at 50 °C, and reactions were monitored at 412 nm.

Enzyme kinetics: Wells comprised 60 µL in total including *Th*AOS (0.3 mg/mL), 0.5 mM DTNB, 0-32 mM amino-acid and 1 mM acetyl-CoA at pH 7.5.

Acyl-CoA screen: Wells comprised 60 µL in total including *Th*AOS (0.3 mg/mL), 0.5 mM DTNB, 16 mM amino-acid and 1 mM acyl-CoA at pH 7.5.

**HPLC Analysis**

Reactions were quenched with 1 volume of acetonitrile and centrifuged to pellet precipitated protein. Reaction mixtures (10 µL) were analysed using a Luna 5µ C18 (2) RP-HPLC column (100 Å, 250x4.60 mm, Phenomenex) at 30 °C under the following conditions: 5% MeCN (0.1% TFA) in $H_2O$ (0.1% TFA) 0-5 min, 5-55% 5-7.5 min, 55% 7.5-17.5 min, 55-5% 17.5-20 min, 5% 20-22.5 min. Integrations were taken from the machine software and converted to concentrations *via* the use of a standard curve.

**LC ESI MS Analysis of Pyrroles**

Samples were prepared in the same manner as those for the HPLC assay, with the exception that reactions were quenched with MeCN. ESI-MS analysis was performed on a Bruker MicroTOF II coupled to an Agilent 1100/1200 Series HPLC. The column used was a Phenomenex Jupiter C18 300 Å column coupled to an ESI source. The LC gradient ran from 5-95% MeCN/$H_2O$ to 95-5% MeCN/$H_2O$ over 10 minutes. The extracted ion chromatograms (EICs) of the target mass was analysed using Bruker DataAnalysis software and data was plotted using Origin 2019.

**Knorr Pyrrole Reaction (KPR) Optimisation**

MAA (0 – 32 mM from 0 – 1.6 M stocks in MeCN, EtOH or DMSO) was incubated with aminoacetone hydrochloride (2 mM) under buffer conditions (20 mM HEPES, 150 mM NaCl, pH 7.5) at 50 °C for 2h and the formation of pyrrole product was determined using HPLC. MeCN was found to be a marginally

superior co-solvent and it was clear that great excess of MAA is required to achieve good conversions of pyrrole under short timescales.

MAA (32 mM from a 1.6 M stock in MeCN) was incubated with aminoacetone (2 mM) under buffer conditions (20 mM HEPES, 150 mM NaCl) at pH intervals of 0.2 for 2h at 50 °C and the final yield of pyrrole was determined using HPLC.

# 4. Preparation of Pyrrole Standards (**1, 5, 21, 25, 26, 28**)

**Methyl 2,4-dimethyl-1H-pyrrole 3-carboxylate (1)** Aminoacetone hydrochloride (0.8 g) was stirred in $H_2O$ (180 mL) with methyl acetoacetate (300 mM from a 1.6 M stock in acetonitrile) at 80 °C under inert atmosphere. pH was monitored and maintained above 9.0 with aqueous NaOH (5 M). When the reaction had run to significant completion after ~4h, the reaction mixture was allowed to cool to RT before cooling on ice. The white precipitate was filtered and dried, giving 0.18 g title compound (15.6% yield).

**$^1$H NMR** (500 MHz, $CDCl_3$): $\delta_H$ 7.91 (1H, broad s, N*H*), 6.37 (1H, s, 2'-C*H*), 3.83 (3H, s, -OC$H_3$), 2.50 (3H, s, -C$H_3$), 2.25 (3H, s, -C$H_3$). **$^{13}$C NMR** (125 MHz, $CDCl_3$): $\delta_C$ 166.9, 135.9, 121.6, 114.1, 110.5, 50.4, 21.2, 14.0, 12.4. **GC-MS**: m/z (EI) 153.11 ($M^+$, 96%), 122.1 (100), 121.1 (50), 93.1 (43).

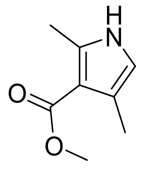


**1**

**Methyl 2-methyl-4-ethyl-1H-pyrrole 3-carboxylate (5)** L-hydroxynorvaline (10 mg) was incubated with purified *E. coli* threonine dehydrogenase (3 mg/mL), $NAD^+$ (2 mM), sodium pyruvate (20 mM), rabbit muscle lactate dehydrogenase (0.05 mg/mL, ammonium sulfate suspension purchased from Sigma Aldrich) and MAA (32 mM from a 3.2 M acetonitrile stock) in HEPES buffer (6 mL, pH 7.5). The reaction mixture was shaken at 37 °C overnight before filtration before extraction with EtOAc (3x10 mL). The crude mixture was purified by flash chromatography (20:1 hexane:EtOAc) to afford 9.6 mg title compound (76.5% yield).

**$^1$H NMR** (500 MHz, $CDCl_3$): $\delta_H$ 7.92 (1H, broad s, N*H*), 6.38 (1H, s, C*H*), 3.82 (3H, s, -OC$H_3$), 2.72 (2H, quart, J=7.5 Hz, -C$H_2$CH$_3$), 2.51 (3H, s, -C$H_3$), 1.02 (3H, t, J=7.5 Hz, -C$H_2$CH$_3$). **$^{13}$C NMR** (125 MHz, $CDCl_3$): $\delta_C$ 166.3, 136.0, 128.8, 113.0, 110.0, 50.4, 20.16, 14.5, 14.2. **GC-MS**: m/z (EI) 167.15 ($M^+$, 100%), 152.1 (62), 122.1 (65), 93.05 (17).

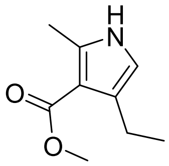


**5**

**Methyl 2-methyl-4-methyl-1H-pyrrole 3-carboxylate (21)** Aminoacetone hydrochloride (0.25 g) was stirred in $H_2O$ (10 mL) with methyl-3-oxopentanoate (320 mM from a 3.2 M acetonitrile stock) at 80 °C under inert atmosphere. pH was monitored and maintained above 9.0 with aqueous NaOH (5 M). When the reaction had run to significant completion after ~4h, the reaction mixture was allowed to cool to RT before extraction with EtOAc (3x 10 mL), drying and concentration. Title compound was purified via flash column chromatography (20:1 hexane:EtOAc) to give a yield of 34 mg (17.7% yield).

**$^1$H NMR** (400 MHz, $CDCl_3$): $\delta_H$ 7.96 (1H, broad s, N*H*), 6.40 (1H, s, 2'-C*H*), 3.82 (3H, s, -C$H_3$), 2.96 (2H, quar, J=7.6 Hz, -C$H_2$CH$_3$), 2.26 (3H, s, -C$H_3$), 1.25 (3H, t, J=7.6 Hz, -CH$_2$C$H_3$). **$^{13}$C NMR** (100 MHz, $CDCl_3$): $\delta_C$ 166.4, 141.7, 121.7, 109.8, 113.8, 50.3, 21.2, 13.3, 12.5. **GC-MS**: m/z (EI) 167.15 ($M^+$, 100%), 152.1 (62), 136.15 (51), 122.1 (65), 93.05 (17). **GC-MS**: m/z (EI) 167.15 ($M^+$, 100%), 152.1 (94), 136.1 (57), 120.1 (50), 135.1 (34), 93.1 (23)

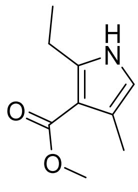


**21**

**Ethyl 2,4-dimethyl-1H-pyrrole-3-carboxylate (25)** was purchased from Fluorochem.

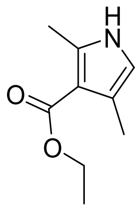


**25**

**2,4-dimethyl-3-acetylpyrrole (26)** Aminoacetone hydrochloride (0.5 g) was stirred in $H_2O$ (20 mL) with acetylacetone (320 mM from a 3.2 M stock in acetonitrile) at 80 °C under inert atmosphere. pH was monitored and maintained above 9.0 with aqueous NaOH (5 M). After 4h the reaction was allowed to cool to RT and subsequently cooled on ice. The light pink precipitate was filtered and dried, giving 91 mg pure compound **27**, an isolated yield of 26.3%.

**$^1$H NMR** (400 MHz, $CDCl_3$): $\delta_H$ 8.04 (1H, broad s, N*H*), 6.39 (1H, s, 2'-C*H*), 2.53 (3H, s, -C$H_3$), 2.46 (3H, s, -C$H_3$), 2.30 (3H, s, -C$H_3$). **$^{13}$C NMR** (100 MHz, $CDCl_3$): $\delta_C$ 195.2, 122.2, 120.9, 114.8, 30.7, 15.3, 13.6. **GC-MS**: m/z (EI) 137.1 ($M^+$, 61%), 122.1 (100)

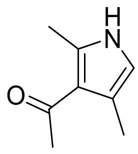


**26**

**3-methyl-4-oxo-4,5,6,7-tetrahydroindole (28)** Aminoacetone hydrochloride (0.25 g) was stirred in $H_2O$ (10 mL) with cyclohexane-1,3-dione (0.3 g) at 80 °C under inert atmosphere. pH was monitored and maintained above 9.0 with aqueous NaOH (5 M). Over time a white precipitate formed and after 4h this was filtered and dried. The white precipitate was filtered and dried, giving 0.12 g pure compound **29**, corresponding to a yield of 72%.

**$^1$H NMR** (400 MHz, $CDCl_3$): $\delta_H$ 8.19 (1H, broad s, N*H*), 6.42 (1H, m, 2'-C*H*), 2.80 (2H, t, J=6.4 Hz, C(O)$CH_2$), 2.50 (2H, t, J=6.4 Hz, C(O)$CH_2CH_2CH_2$), 2.32 (3H, d, J=1.1 Hz, $CH_3$), 2.14 (2H, q, J=6.4 Hz, C(O)$CH_2CH_2$). **$^{13}$C NMR** (100 MHz, $CDCl_3$): $\delta_C$ 194.3, 119.4, 118.9, 115.9, 38.4, 24.0, 23.0, 11.6. **GC-MS**: m/z (EI) 149.15 ($M^+$, 98%), 121.1 (88), 93.1 (100).

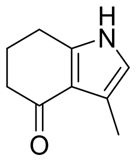

**28**

## 5. Scaled-Up Chemo-Enzymatic Synthesis of Pyrrole (**1**)

**Synthesis and Isolation of Pyrrole (1)**

Acetyl-CoA (100 mg, 0.12 mmol) was added to a buffered solution (100 mM HEPES, 150 mM NaCl, pH 7.5, 20 mL) of methyl acetoacetate (32 mM), glycine (16 mM) and *Th*AOS (1 mgmL$^{-1}$). The solution was stirred at 70 °C under inert atmosphere for 2 hours. When HPLC indicated that the reaction was mostly complete, protein was precipitated with a brief boiling step before precipitation by centrifugation. The reaction mixture was then extracted with EtOAc (3 × 20 mL) before drying with $MgSO_4$ and solvent removal by rotary evaporation. The title compound was purified by flash silica chromatography (10:1 hexane:EA) to afford a pale brown solid (16.4 mg, 0.107 mmol, 86.6%). Analytical data matched that of a previously prepared synthetic standard.

## 6. Formation of Pyrroles by the coupled *Th*AOS/KPR cascade

**Formation of Pyrroles (1-20) using MAA as the KPR reagent**

Amino-acid (32 mM) was incubated with *Th*AOS (1 mg mL$^{-1}$), acyl-CoA (2 mM) and MAA (32 mM from a 3.2 M stock in MeCN) under buffer conditions (100 mM HEPES, 150 mM NaCl, pH 7.5) at 70 °C for 2 hours with shaking at 250 rpm. Reactions were quenched by addition of one volume of MeCN and centrifugation at 13,000xg for 10 minutes to pellet precipitated protein, before analysis of the mixture by LC ESI-MS analysis as described above.

**Formation of Pyrroles (5, 21, 25, 26 and 28) using alternative KPR reagents**

Gly (32 mM) was incubated with ThAOS (1 mg mL$^{-1}$), acyl-CoA (2 mM) and either the β-keto ester (BKE) or β-keto ketone (BKK) (32 mM from a 3.2 M stock in MeCN, or a 3.2 M stock in $H_2O$ in the case of 1,3-cyclohexanedione, **28b**) in buffer (100 mM HEPES, 150 mM NaCl, pH 7.5) at 70 °C for 2 hours (apart from entry **21*** for 4 hrs) with shaking at 250 rpm. Reactions were quenched by the addition of one volume of MeCN and centrifugation at 13,000xg for 10 minutes to pellet precipitated protein, before analysis of the mixture by HPLC as described above.

**Formation of Pyrrole (1) by a Biocatalytic Cofactor Recycling System**

The *Th*AOS (1 mg mL$^{-1}$) was incubated with Gly (32 mM), MAA (32 mM from a 3.2 M stock in MeCN), sodium benzoate (1 mM), CoASH or acyl-CoA (1 mM), sodium acetate (32 mM), ATP (8 mM), PPase (0 or 1 mg mL$^{-1}$) and ACS (1 mgmL-1 ) in HEPES buffer (100 mM, 150 mM NaCl pH 7.5) at a final volume of 200 µL in an Eppendorf tube. Reactions were performed in a Grant-Bio 24-well thermoshaker with shaking at 250 rpm at 37 °C for 16h. Reactions were initiated by addition of ATP. Reactions were terminated by addition of 1 volume of MeCN, and centrifugation for 10 minutes at 13,000 g. The supernatant was then analysed by HPLC.

# 7. X-Ray Data Collection and Model Refinement

**Protein Crystallisation**

Crystallisation of recombinant *Th*AOS was initially screened using commercial kits (Molecular Dimensions and Hampton Research). Protein concentration was 20-25 mg $mL^{-1}$. The drops, comprising 0.1 or 0.2 µL of protein solution plus 0.1 µL of reservoir solution, were set up using a Mosquito crystallisation robot (SPT Labtech). The experiments were incubated at 20 °C. Initial hits were of good size, single and could be directly tested. Whilst hits were found in Index (Hampton Research), three conditions were found in Morpheus (A8, A12 and C8, Molecular Dimensions) to lead to alternative crystal space groups. *Th*AOS crystallised in P1 (30 mM sodium nitrate, 30 mM sodium phosphate, 30 mM ammonium sulfate, 100 mM HEPES/MOPS pH 7.5, 12.5% (w/v) PEG1000 and 12.5% (w/v) PEG3350), $P2_1$ (30 mM magnesium chloride, 30 mM calcium chloride, 100 mM HEPES/MOPS pH 7.5, 12.5% (w/v) PEG1000 and 12.5% (w/v) PEG3350) and in $P2_12_12_1$ (30 mM magnesium chloride, 30 mM calcium chloride, 100 mM Tris/bicine pH 8.5, 12.5% (v/v) MPD, 12.5% (w/v) PEG1000 and 12.5% (w/v) PEG3350). The samples did not require optimisation of additional cryo-protection.

**Data Collection, Structure Solution, Model Building, Refinement and Validation**

Diffraction data were collected at the synchrotron beamline I03 of Diamond light source (Didcot, UK) at a temperature of 100 K.

The $P2_1$ data set was integrated with autoPROC[1] and the others with XIA2[2] using XDS[3] and scaled with Aimless.[4]

The space groups were confirmed with Pointless.[5]

The phase problem was solved by molecular replacement with Phaser[6] using PDB file 3TQX as search model.[7]

The models were first improved with an automated model building round of Buccaneer[8] and were refined with refmac.[9]

Manual model building with COOT[10] was intercalated between refinement rounds.

The models were validated using COOT and Molprobity.[11] Other software used were from CCP4 cloud[12] and CCP4 suite.[13]

Figures were made with Chimerax.[14] Data collection processing and refinement statistics are presented in Table **S4**.

# 8. Mechanism of AOS Enzymes

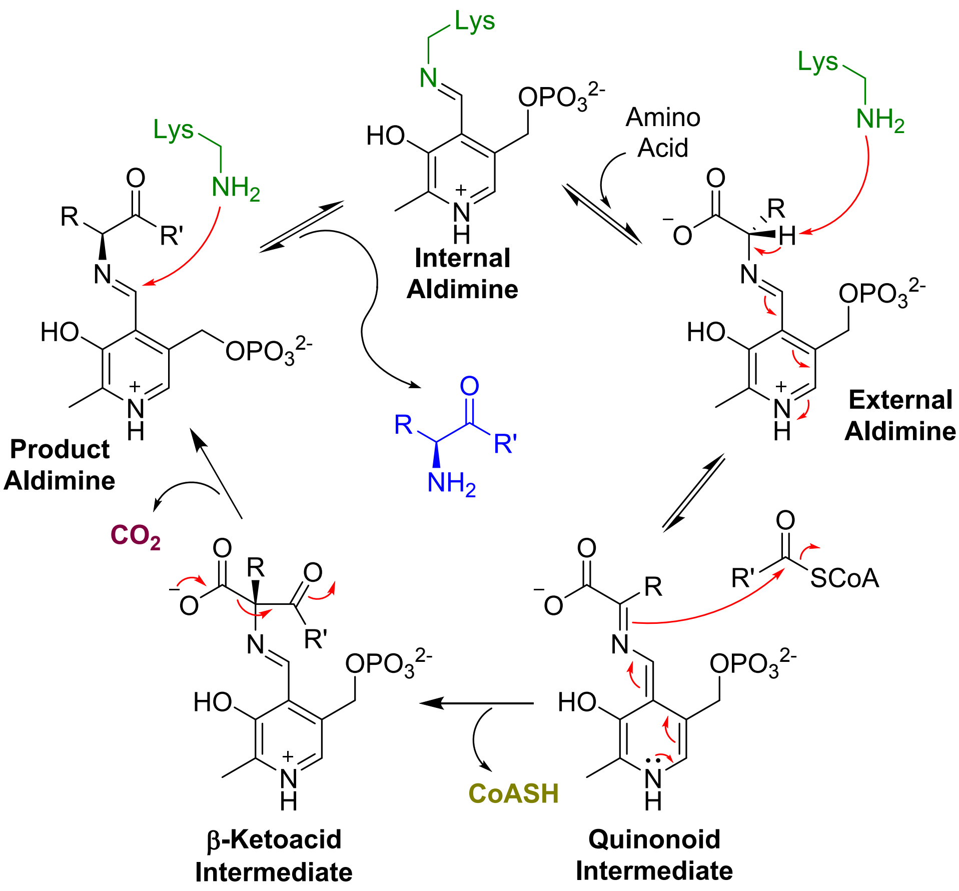


***Figure S1: The proposed catalytic cycle of the α-oxoamine synthases (AOSs).***[15-17] *The resting state of the enzyme comprises an internal-aldimine species in which the PLP cofactor is covalently linked to an active site Lys residue on the enzyme scaffold via a Schiff base linkage. The first chemical step of the cycle is transimination by the amino-acid substrate to generate a PLP: external-aldimine, in which the PLP cofactor is no longer covalently bound to the enzyme and the active site Lys is freed. The same Lys deprotonates the amino-acid* $C_{\alpha}$*, with the departing σ-bond electron density being accepted by the PLP electron sink. In most cases this deprotonation is permitted only upon the Michaelis binding of the acyl-CoA substrate to the enzyme. This step generates a reactive quinonoid-like intermediate species, which subsequently acts as a carbanion to generate a new C-C bond by attacking the acyl-CoA thioester carbon, with loss of CoASH. The new α-amino-β-ketoacid species is decarboxylated inside the enzyme active site and reprotonated with retention of stereochemistry, yielding a product-aldimine which is subsequently transaminated by the catalytic Lys residue to release the α-aminoketone product.*

# 9. Purification Recombinant Biocatalysts (*Th*AOS and *Ec*ACS)

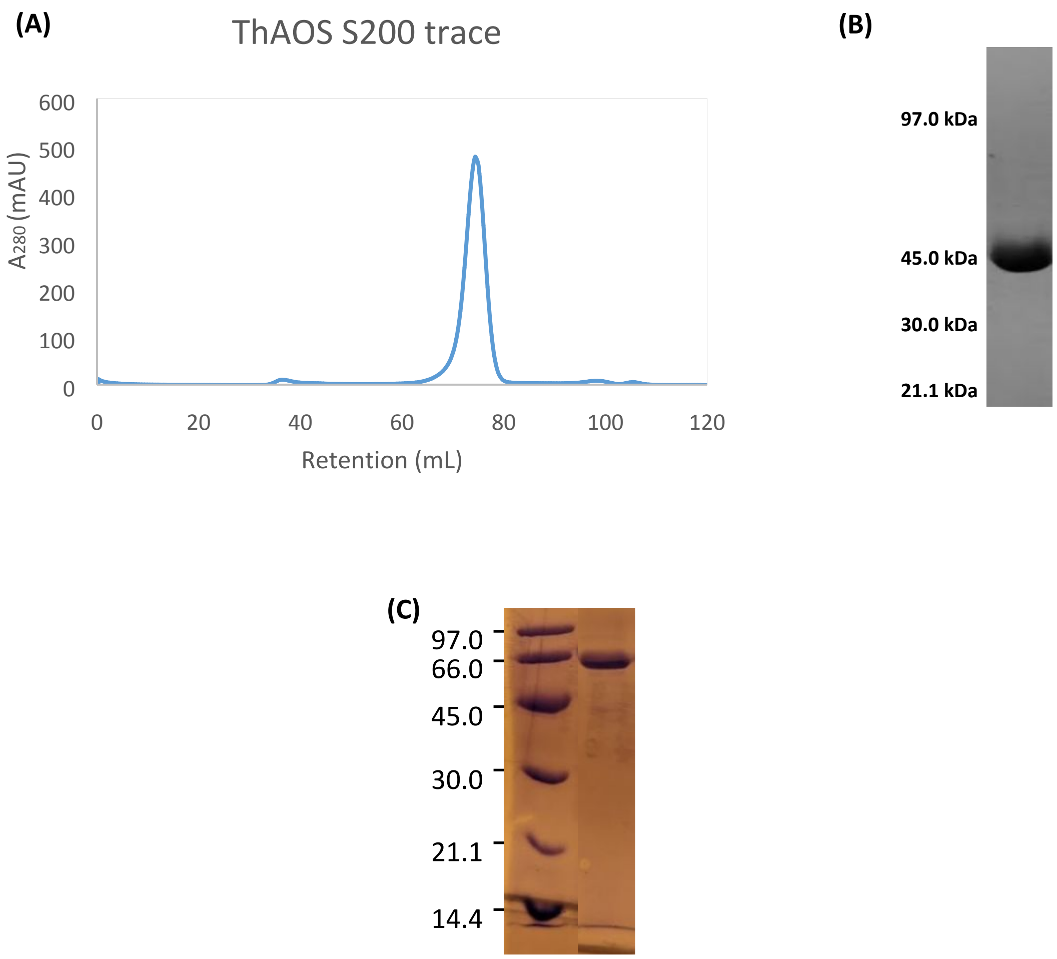


***Figure S2: Purification of recombinant biocatalysts.*** *(A) After* $Ni^{2+}$*-IMAC, the pooled fractions were analysed by* Th*AOS Size Exclusion Chromatography (SEC) on Sephadex S200 HR. The chromatogram shows a typical purification of recombinant* Th*AOS used for assays and crystal trials. (B) SDS-PAGE analysis of pure ThAOS at ~45 kDa. (C) The recombinant* Ec*ACS used in the cofactor recycling study was isolated via a similar two-step purification procedure and gave a pure band at ~66 kDa.*

# 10. Assay of Recombinant *Th*AOS

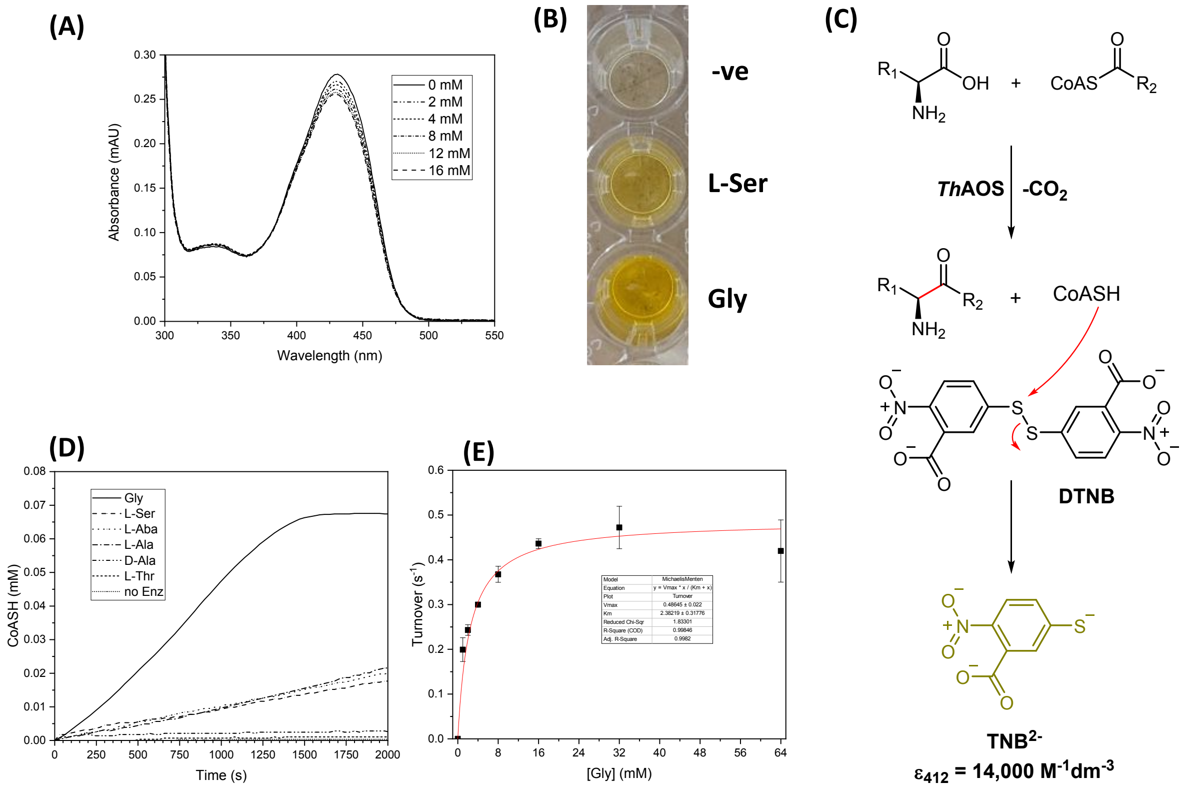


***Figure S3: Assay of* Th*AOS.*** *(A) UV-vis absorption spectrum of purified, recombinant* Th*AOS, including spectral changes which occur upon the titration of Gly. (B) Image of the DTNB assay in the cases of no amino-acid (negative control), L-Ser and Gly. Addition of the active substrate changes the color of the respective wells to yellow due to the formation of the brightly colored TNB$^{2-}$ anion. (C) Reaction scheme of the DTNB colorimetric assay. The Claisen-like condensation between an α-amino-acid and an acyl-CoA thioester catalysed by* Th*AOS releases CoASH as a free thiol, which then reacts* in situ *with the colourless assay substrate DTNB to release the brightly-colored yellow anion, TNB$^{2-}$. (D) Outcome of the initial DTNB careen of amino-acid substrates (Gly, L-Ser, L-Aba, L-Ala, D-Ala, L-Thr) with* Th*AOS and acetyl-CoA. (E) Example Michaelis-Menten kinetic characterisation of* Th*AOS with amino-acid substrate Gly.*

# 11. Substrate Scope *of ThAOS*

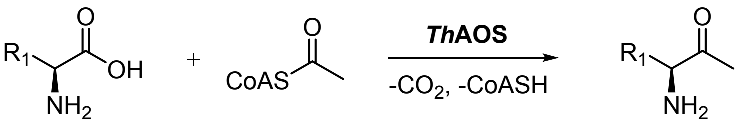


| Substrate | $k_{cat}$ ($min^{-1}$) | $K_M$ (mM) | $k_{cat}/K_M$ ($min^{-1}mM^{-1}$) |
|---|---|---|---|
| L-Aba | 3.68 ± 0.58 | 24.2 ± 6.28 | 0.15 |
| L-Ala | 6.24 ± 0.41 | 1.63 ± 0.64 | 3.82 |
| Gly | 29.2 ± 1.85 | 2.38 ± 0.32 | 12.27 |
| L-Ser | 6.78 ± 0.32 | 5.12 ± 0.85 | 1.32 |
| Acetyl-CoA | 30.1 ± 3.69 | 0.33 ± 0.09 | 91.2 |

***Table S1: The kinetic parameters of* Th*AOS.*** *This was carried out with four amino-acid substrates (L-Aba, L-Ala, Gly and L-Ser) and acetyl-CoA as the acyl-thioester, and activity measured at 50 °C using the DTNB colorimetric assay (three replicates).*

| Amino-Acid | CoA-Thioester | Specific Turnover ($min^{-1}$) |
|---|---|---|
| L-Ala | Acetyl- | 15.0 ± 1.08 |
| L-Ala | Propionyl- | 7.02 ± 0.30 |
| L-Ala | Butyryl- | 3.78 ± 0.58 |
| L-Ala | Hexanoyl- | 3.54 ± 0.042 |
| L-Ala | Octanoyl- | 1.74 ± 0.3 |
| Gly | Acetyl- | 27.40 ± 1.74 |
| Gly | Propionyl- | 12.05 ± 0.12 |
| Gly | Butyryl- | 2.88 ± 0.53 |
| Gly | Hexanoyl- | 4.68 ± 0.18 |
| Gly | Octanoyl- | 1.26 ± 0.06 |
| L-Ser | Acetyl- | 14.75 ± 0.48 |
| L-Ser | Propionyl- | 0.36 ± 0 |
| L-Ser | Butyryl- | 0.18 ± 0.018 |
| L-Ser | Hexanoyl- | 1.26 ± 0.12 |
| L-Ser | Octanoyl- | 0.72 ± 0.30 |
| L-Aba | Acetyl- | 5.52 ± 0.78 |
| L-Aba | Propionyl- | 1.02 ± 0.36 |
| L-Aba | Butyryl- | 0.54 ± 0.012 |
| L-Aba | Hexanoyl- | 0.36 ± 0.06 |
| L-Aba | Octanoyl- | 1.86 ± 0.60 |

***Table S2: Summary of the specific activities of* Th*AOS for all active amino-acid/substrate pairings.*** *Data were measured at 50 °C in the presence of quasi-saturating substrate concentrations (16 mM amino-acid and 1 mM acyl-CoA thioester). Turnover measured with the DTNB colorimetric assay (three replicates).*

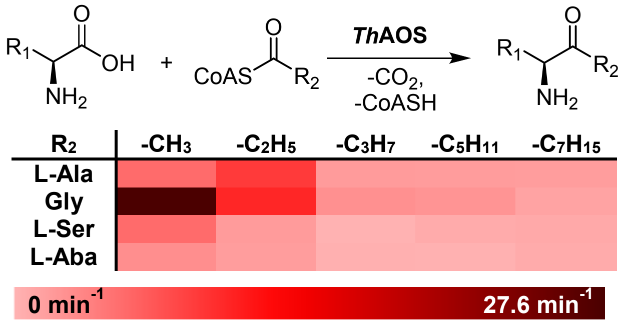


***Table S3. Summary of the Chain length selectivity of* Th*AOS***. *Conditions: ThAOS (1 mgmL*$^{-1}$*), amino-acid (16 mM), acyl-CoA (1 mM), DTNB (0.5 mM), 60 µL, 50 °C.*

| *Enzyme* | *Amino-acid* | *Acyl-CoA* | *Pathway* |
|---|---|---|---|
| *AONS*[18] | L-Ala | Pimeloyl- | Biotin |
| *KBL*[19] | Gly | Acetyl- | L-Thr |
| *ALAS*[20] | Gly | Succinyl- | Heme |
| *SPT*[21] | L-Ser | Long-chain (C12-C20) | Sphingolipids |
| *CqsA*[22] | L-SAM, L-Aba, Gly | Decanoyl- | Quorum sensing |
| *SxtA*[23] | L-Arg | Straight-chain (C2-C6), $Pr^i$, $Bu^i$, benzoyl-, cyclopentyl- | Saxitoxin |
| *TamD*[24] | L-Ser | 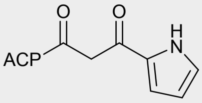 | Tambjamine YP1 |
| *PigH/RedN*[25] | L-Ser | 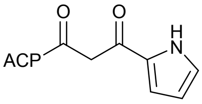 | Prodigiosins |
| *RedL*[26] | Gly | 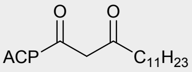 | Prodigiosins |
| *Fum8P*[27] | L-Ala, Gly | Octadecanoyl-, decorated octadecanoyl- | Fumonisins |
| *KtmB*[28] | L-Phe, L-Tyr, L-Trp | 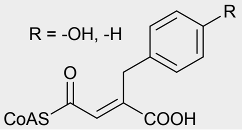 | Ketomemicins |
| *PqrA*[29] | L-Phe, L-Tyr, L-Met | Succinyl- | Perquinolones |
| *PapD*[30] | L-4APhe, L-His, L-Phe, L-Trp, L-Tyr | Acetyl- | Pyrazines |
| *CuaB*[31] | L-Ala | 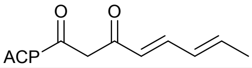 | Curvulamines |
| *Alb29*[32] | L-Glu | 7× medium chain examples (C4-C7) | Albogrisins |
| *BioWF*[33] | L-Ala, Gly, L-Ser | 9× medium chain examples (C6-C9) with L-Ala | Biotin |
| *VsAOS*[34] | L-Tyr | Dodecanoyl-CoA | Vitroprocines |

***Table S4: Summary of the currently known AOS enzymes, their substrate scopes and the biosynthetic pathways they are involved in. Representative references (18-34) with details of each AOS are cited.***

# 12. Thermal stability of *Th*AOS

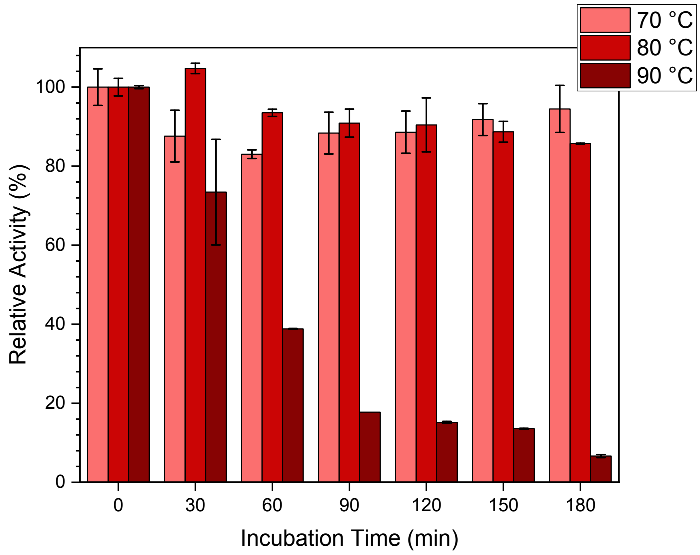


***Figure S4: Thermal stability of*** **Th*****AOS with Gly and acetyl-CoA as a model reaction system.*** Th*AOS was incubated at 70, 80 and 90 °C respectively for 0-180 minutes and the residual activity was determined using the DTNB assay. The* Th*AOS biocatalyst was stable to incubation at 80 °C for 3 hours and retained some activity after prolonged incubation at 90 °C.*

# 13. Optimisation of the Formation of Pyrrole **1** by the chemo-biocatalytic *Th*AOS-KPR cascade.

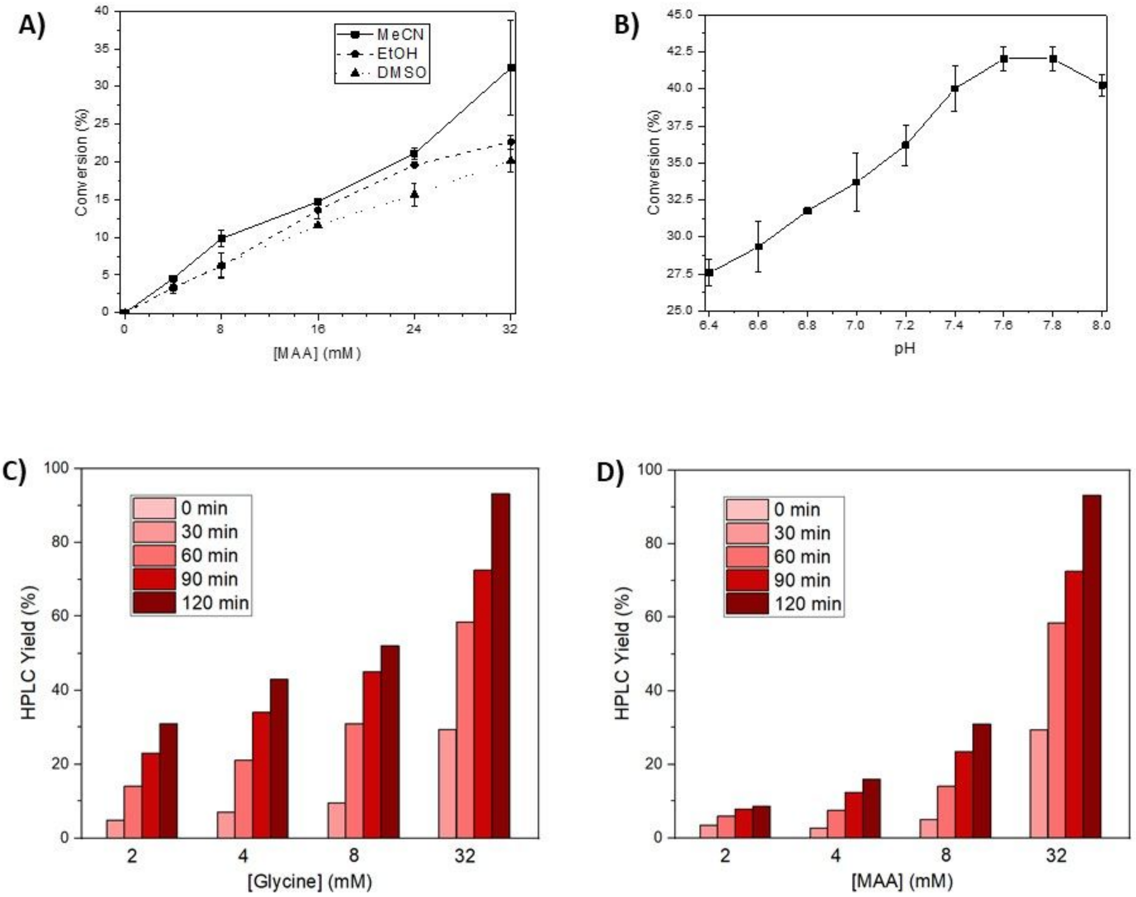


***Figure S5: Optimisation (solvent, pH and biocatalyst loading) of the chemo-biocatalytic* Th*AOS/Knorr pyrrole reaction to form pyrrole 1. (A)*** *Cosolvent was necessary due to the low solubility of MAA. Three cosolvents were screened, as well as [MAA]. MeCN performed marginally better than EtOH or DMSO and increasing [MAA] improved conversion after 2 hrs at 50 ˚C.* ***(B)*** *Optimisation of pyrrole 1 formation peaked at pH 7.6-7.8 after 2 hrs at 50 ˚C.* ***(C)*** *Optimisation of the ThAOS/KPR cascade towards pyrrole* ***1*** *with varying concentrations of the ThAOS substrate glycine (2, 4, 8, 32 mM). The % hplc yield reaches >90% after 2hrs at 70 °C.* ***D)*** *Optimisation of the ThAOS/KPR cascade towards pyrrole* ***1*** *with varying concentrations of KPR reactant MAA (2, 4, 8, 32 mM). The % hplc yield reaches >90% after 2hrs at 70 °C.*

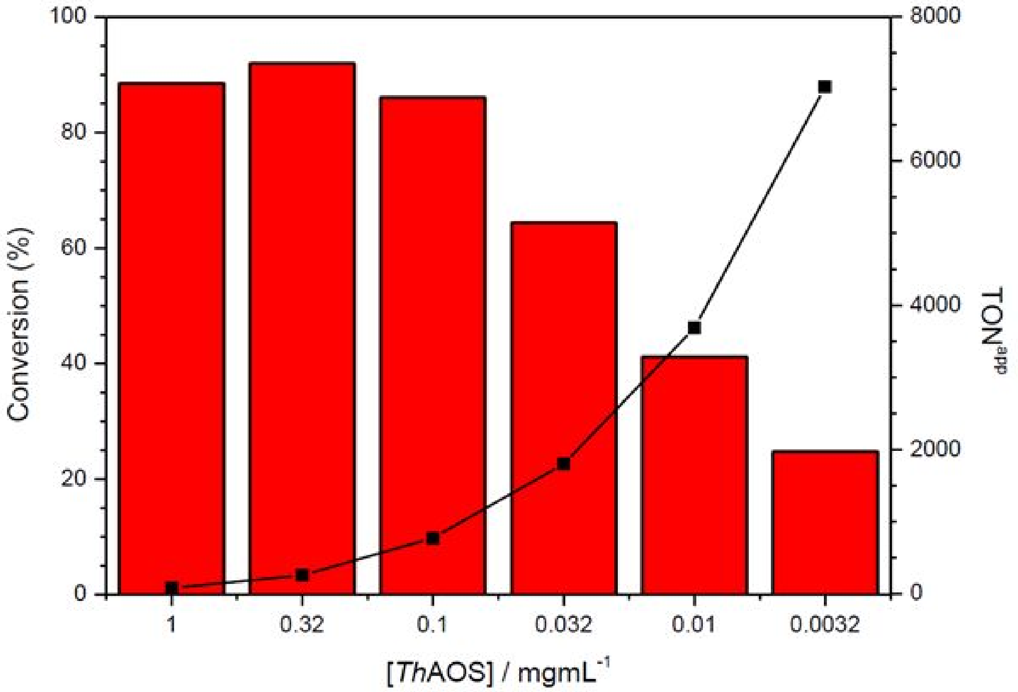


***Figure S6: Screening of [ThAOS] biocatalyst loading for the Gly and acetyl-CoA substrates.*** *The* [*Th*AOS] *could be reduced to as low as 0.1 mgmL$^{-1}$ with 1 mgmL$^{-1}$ selected as the concentration used for development of the cascade with the KPR. The left hand side shows % conversion with red bars and the right hand side shows the apparent turnover number (TON$^{app}$), black boxes connected by a line to show the upward trend from <100 to >7000 TON$^{app}$.*

## 14. Detection of Pyrroles Products (**1-20**) by EIC LC ESI-MS.

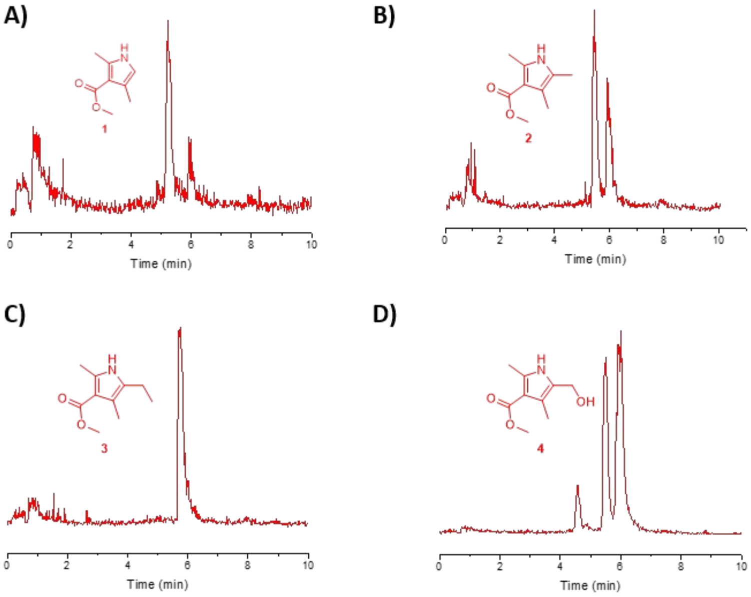


***Figure S7: LC-ESI-MS of the chemo-enzymatic cascade towards pyrrole products 1-4**. Extracted ion chromatograms (EICs) of the pyrrole product ions A) **1** M+H$^+$ $C_8H_{11}NO_2$ m/z = 154.0863, B) **2** M+H$^+$ $C_9H_{13}NO_2$ m/z = 168.1011, C) **3** M+H$^+$ $C_{10}H_{15}NO_2$ m/z = 182.1150, D) **4** M-$H_2O$+H$^+$ $C_9H_{13}NO_3$ m/z = 166.0635).*

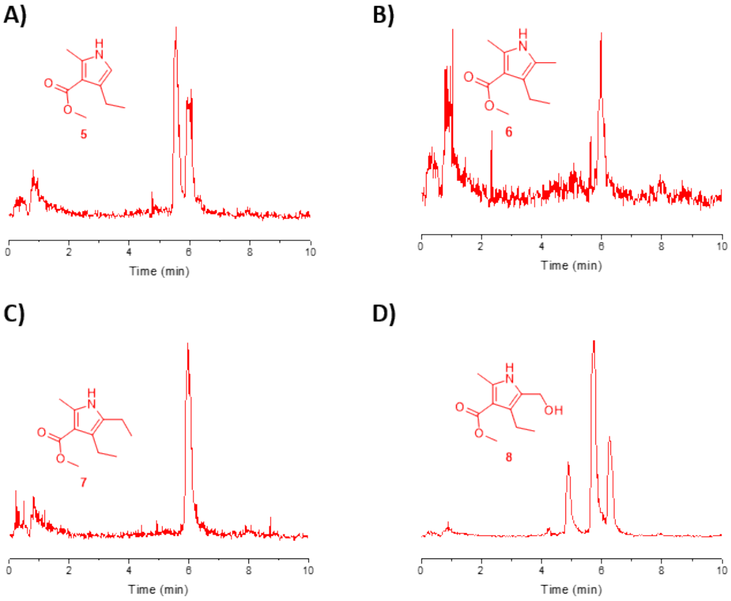


***Figure S8: LC-ESI-MS of the chemo-enzymatic cascade towards pyrrole products 5-8**. EICs of the pyrrole product ions: A) **5** M+H$^+$ $C_9H_{13}NO_2$ m/z = 168.1004, B) **6** M+H$^+$ $C_{10}H_{15}NO_2$ m/z = 182.1152), C) **7** M+H$^+$ $C_{11}H_{17}NO_2$ m/z = 196.1326, D) **8** M-$H_2O$+H$^+$ $C_{10}H_{15}NO_3$ m/z = 180.1019).*

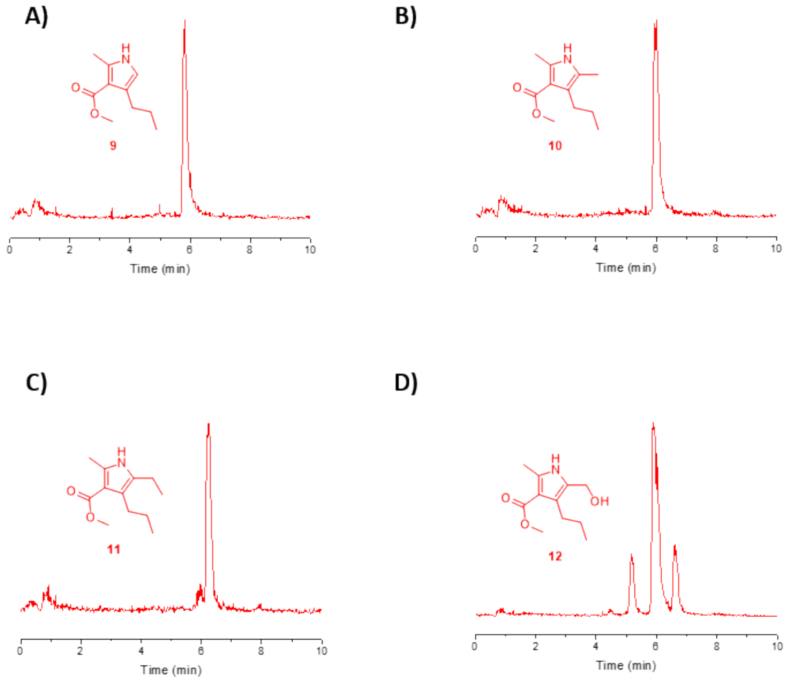


***Figure S9: LC-ESI-MS of the chemo-enzymatic cascade towards pyrrole products 9-12***. *EICs of the pyrrole product ions: A)* ***9*** *M+H$^+$ $C_{10}H_{15}NO_2$ m/z = 182.1192, B)* ***10*** *M+H$^+$ $C_{11}H_{17}NO_2$ m/z = 196.1339, C)* ***11*** *M+H$^+$ $C_{12}H_{19}NO_2$ m/z = 210.1489, D)* ***12*** *M-$H_2O$+H$^+$ $C_{11}H_{17}NO_3$ m/z = 194.1176).*

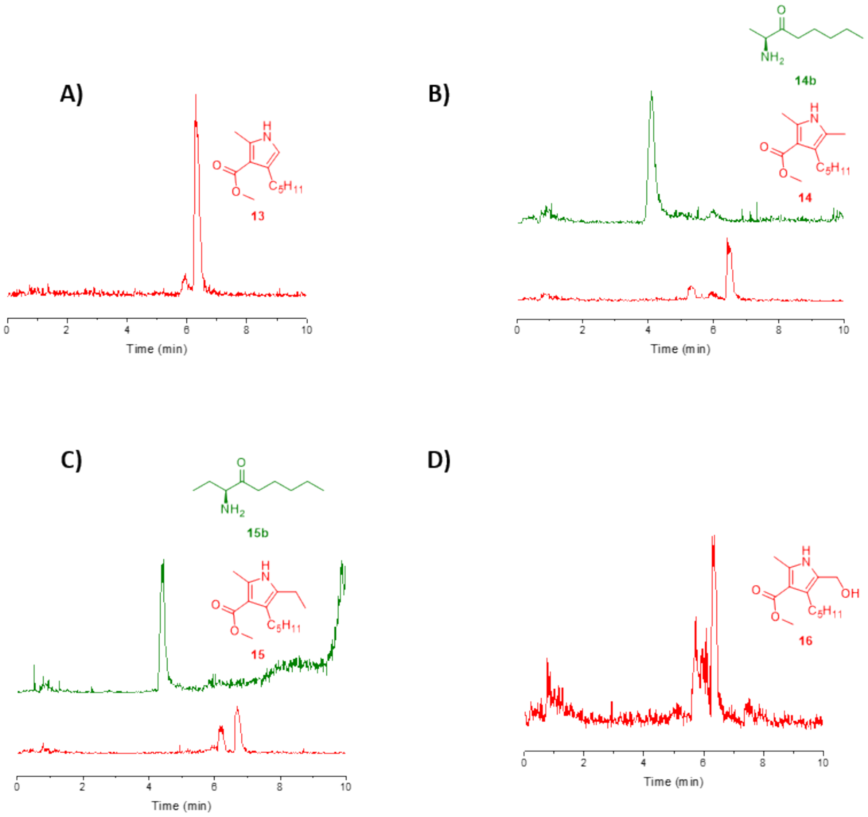


***Figure S10: LC-ESI-MS of the chemo-enzymatic cascade towards pyrrole products 13-16***. *EICs of the pyrrole product ions: A)* ***13*** *M+H$^+$ $C_{12}H_{19}NO_2$ m/z = 210.1469, B)* ***14*** *M+H$^+$ $C_{13}H_{21}NO_2$ m/z = 224.1699, C)* ***15*** *M+H$^+$ $C_{14}H_{23}NO_2$ m/z = 238.1802, D)* ***16*** *M-$H_2O$+H$^+$ $C_{13}H_{21}NO_3$ m/z = 222.1509) are shown in red. The EICs of the observed $\alpha$-aminoketone intermediate ions B)* ***14b*** *M+H$^+$ $C_8H_{17}NO$ m/z = 144.1413, C)* ***15b*** *M+H$^+$ $C_9H_{19}NO$ m/z = 158.1529) are included.*

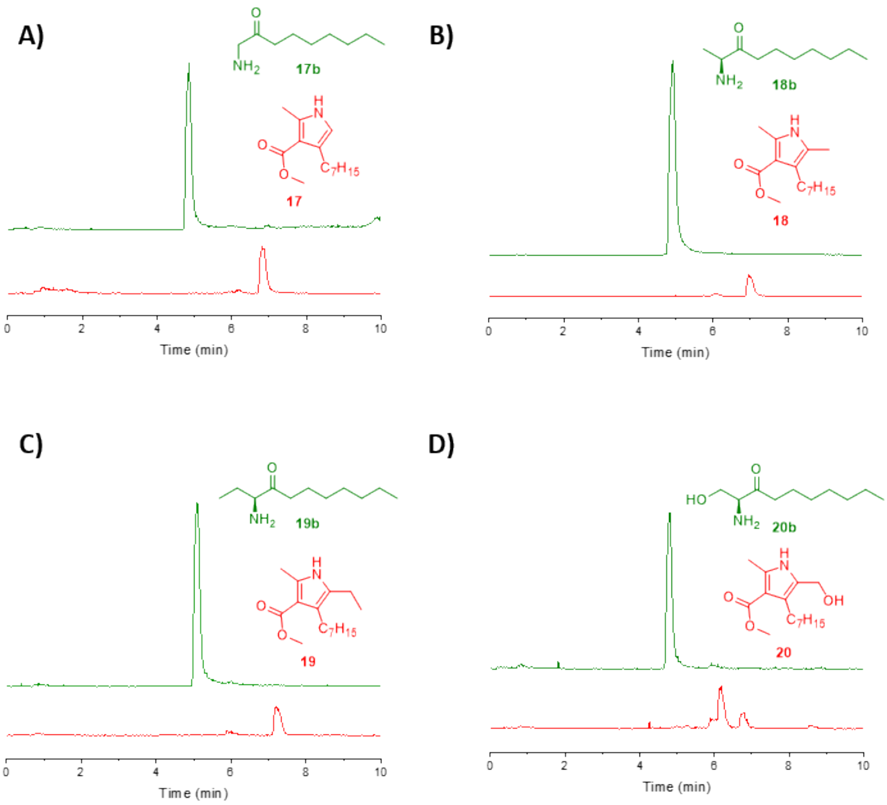


***Figure S11: LC-ESI-MS of the chemo-enzymatic cascade towards pyrrole products 17-20**. EICs of the pyrrole product ions, A) **17** M+H$^+$ $C_{14}H_{23}NO_2$ m/z = 238.1802, B) **18** M+H$^+$ $C_{15}H_{25}NO_2$ m/z = 252.1974, C) **19** M+H$^+$ $C_{16}H_{27}NO_2$ m/z = 266.2115, D) **20** M-H$_2$O+H$^+$ $C_{15}H_{25}NO_3$ m/z = 250.1803) are show in red. The EICs of the observed $\alpha$-aminoketone intermediate ions A) **17b** M+H$^+$ $C_9H_{19}NO$ m/z = 158.1525, B) **18b** M+H$^+$ $C_{10}H_{21}NO$ m/z = 172.1706, C) **19b** M+H$^+$ $C_{11}H_{23}NO$ m/z = 186.1850, D) **20b** M+H$^+$ $C_{10}H_{21}NO_2$ m/z = 188.1656) are included.*

## 15. Analysis of Pyrroles (**1, 5, 21, 25, 26** and **28**) by HPLC.

**A)**

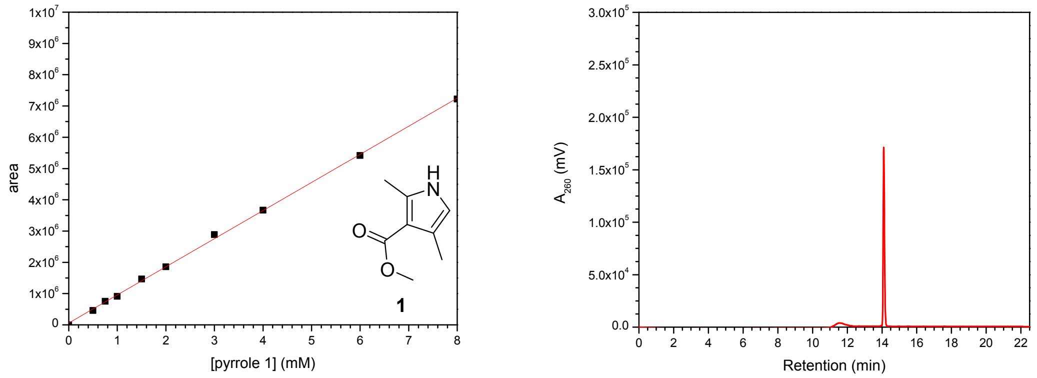


**B)**

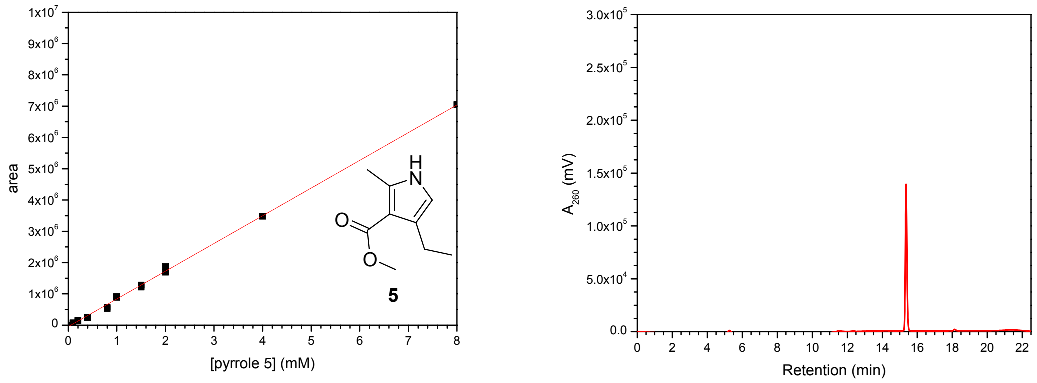


**C)**

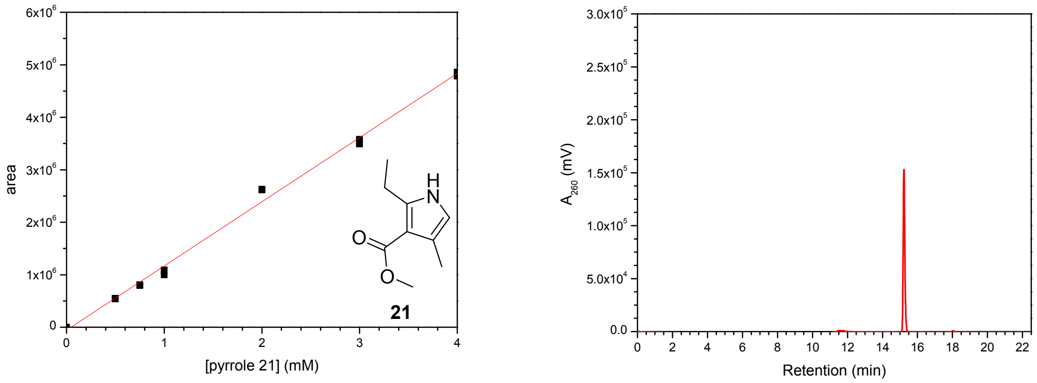

**D)**

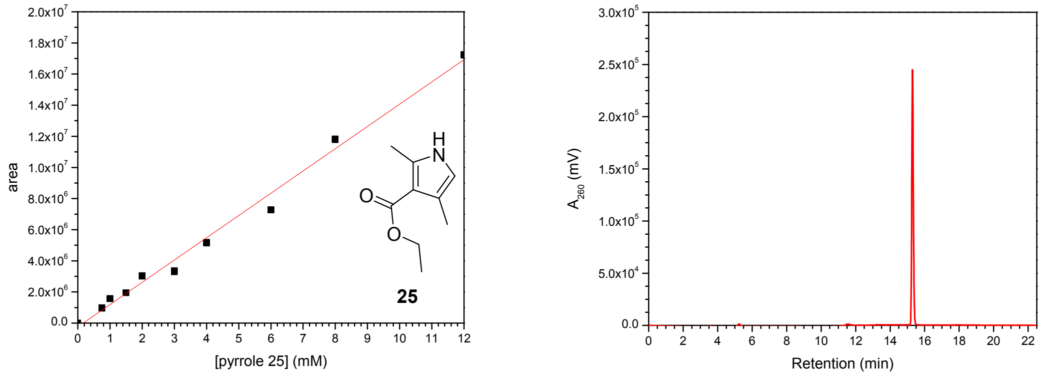


**E)**

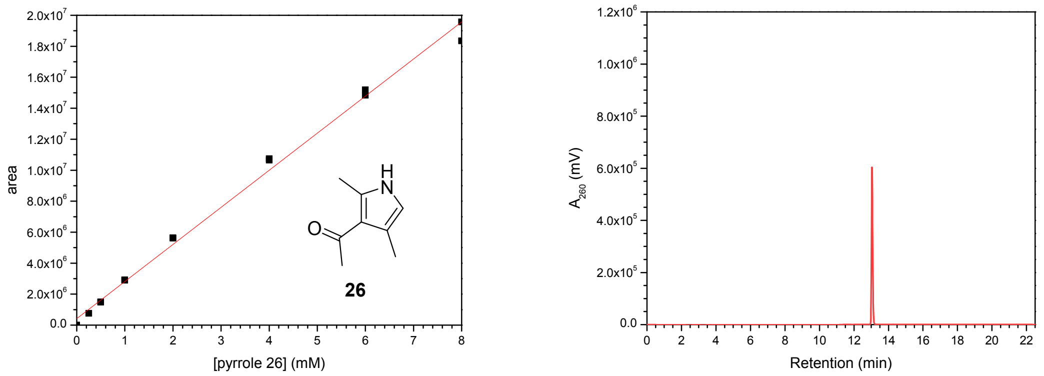


**F)**

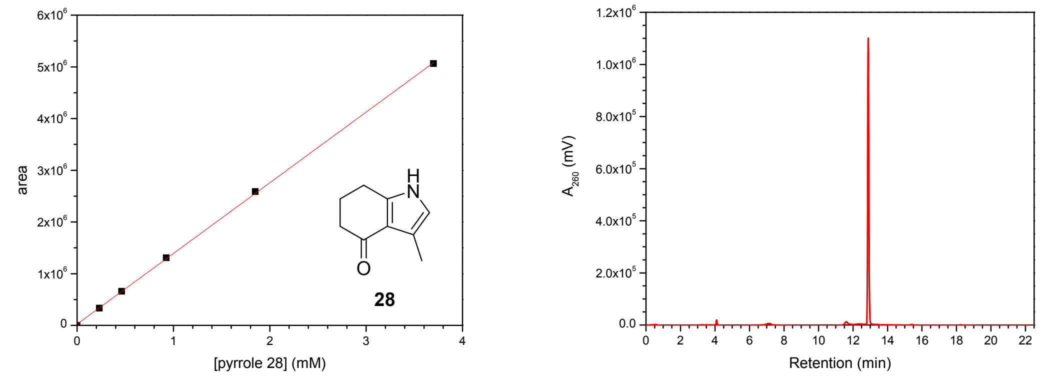


***Figure S12: Pyrrole standard curves.*** *HPLC calibrations for pyrrole products using a $C_{18}$ RP column. The left hand side shows the HPLC response due to injection of 10 ul of a stock standard solution in 100% MeCN. For* ***A) 1*** *(0.5 to 8.0 mM), for* ***B) 5*** *(0.1 to 8.0 mM), for* ***C) 21*** *(0.5 to 4.0 mM), for* ***D) 25*** *(0.75 to 12.0 mM), for* ***E) 26*** *(0.25 to 8.0 mM) ), for* ***F) 28*** *(0.23 to 3.75 mM). The right hand side shows a typical injection (from 1 mM stock).*

# 16. $^{1}H$ and $^{13}C$ NMR Spectra for Pyrroles (**1, 5, 21, 25, 26, 28**).

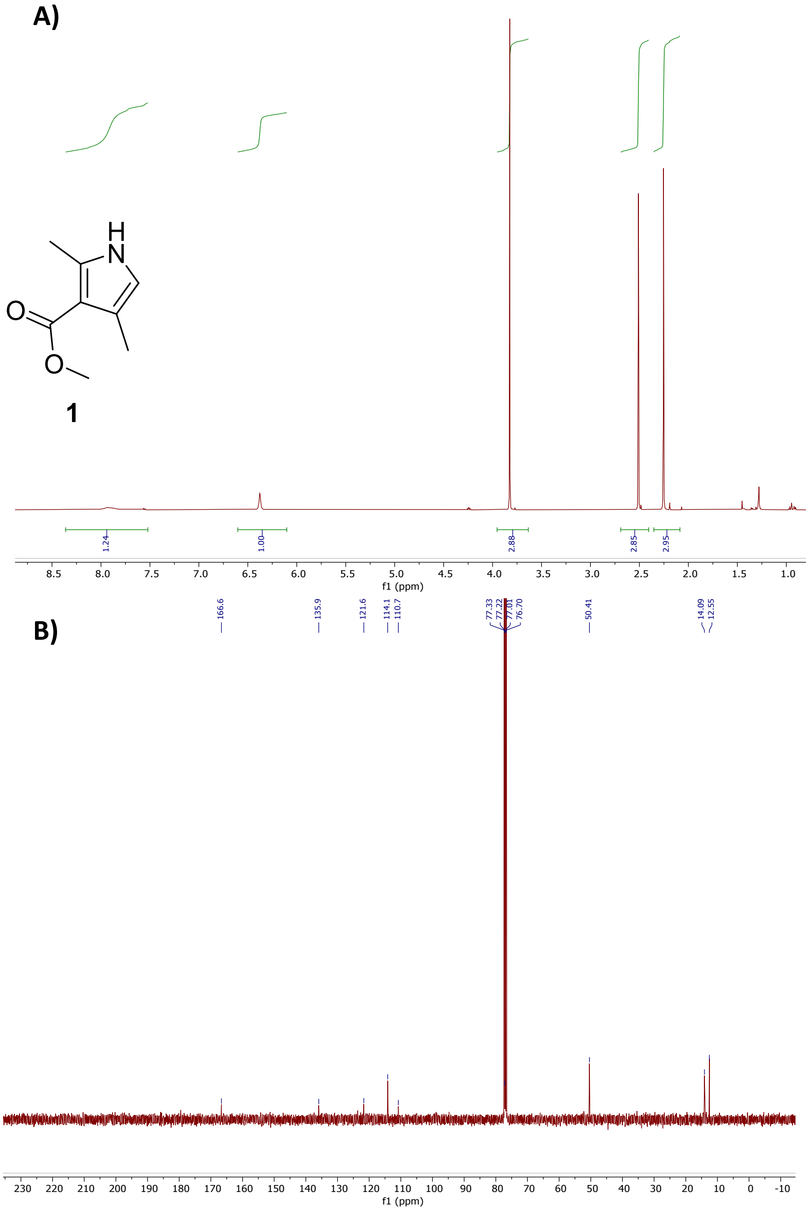


***Figure S13: Isolation and purification of pyrrole 1.*** *NMR analysis of pyrrole 1 isolated from scaled-up ThAOS synthesis. A) $^{1}H$ NMR (500 MHz) and B) $^{13}C$ NMR (125 MHz).*

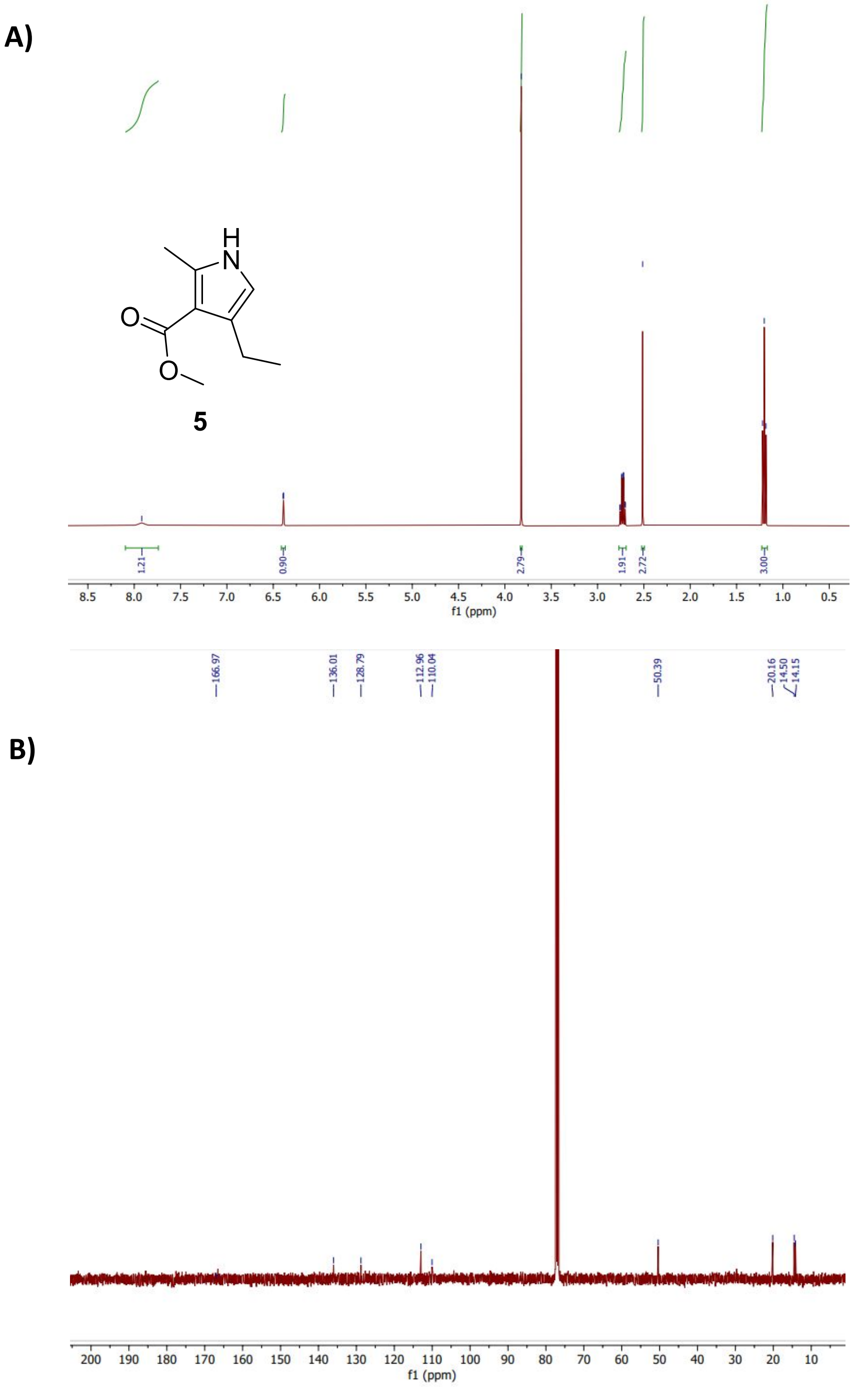


***Figure S14: NMR analysis of pyrrole 5 isolated from chemical synthesis.*** *A) $^{1}H$ NMR (500 MHz) and B) $^{13}C$ NMR (125 MHz).*

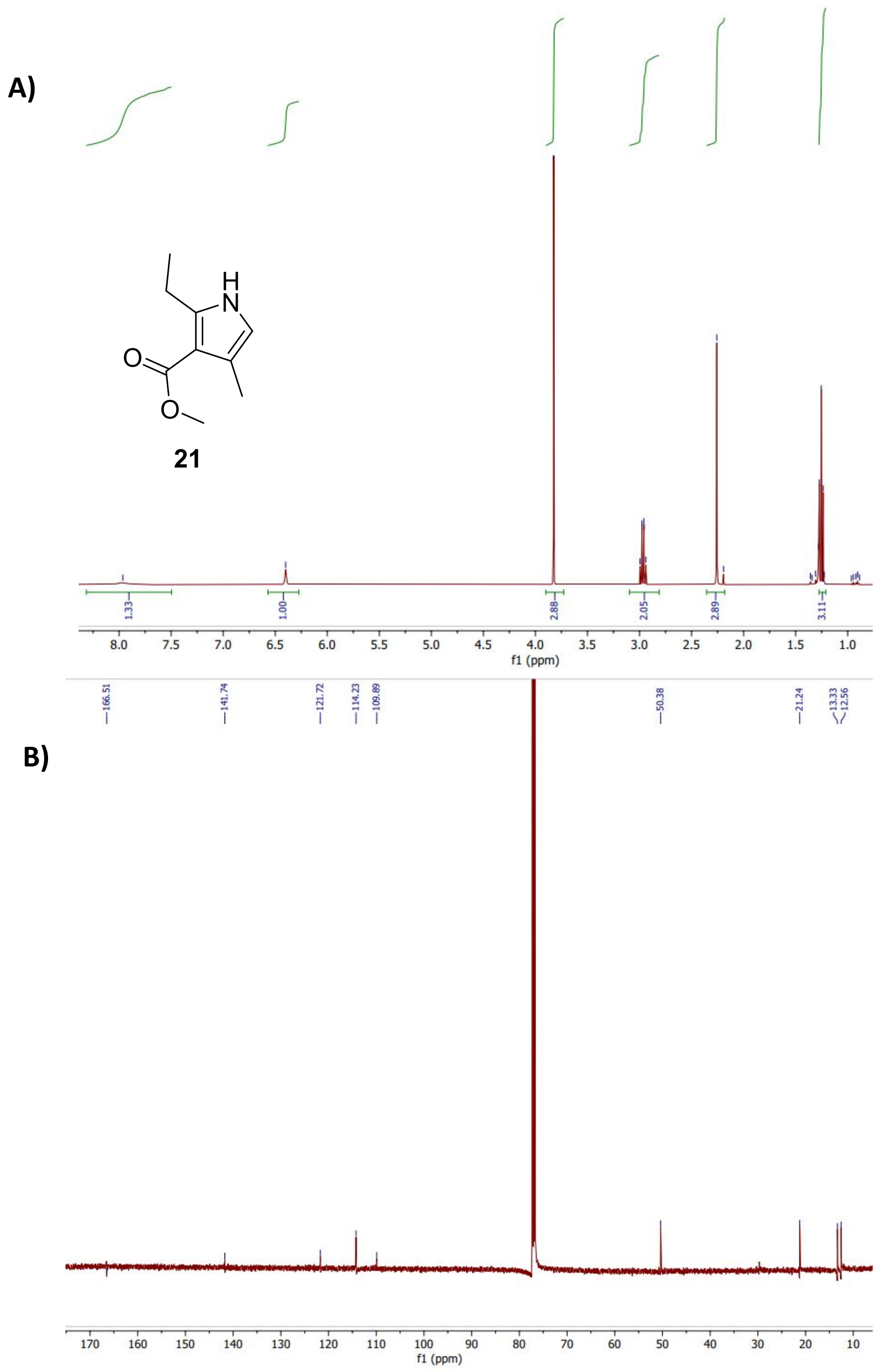


***Figure S15**: NMR analysis of pyrrole **21** isolated from chemical synthesis. A) $^{1}$H NMR (400 MHz) and B) $^{13}$C NMR (100 MHz).*

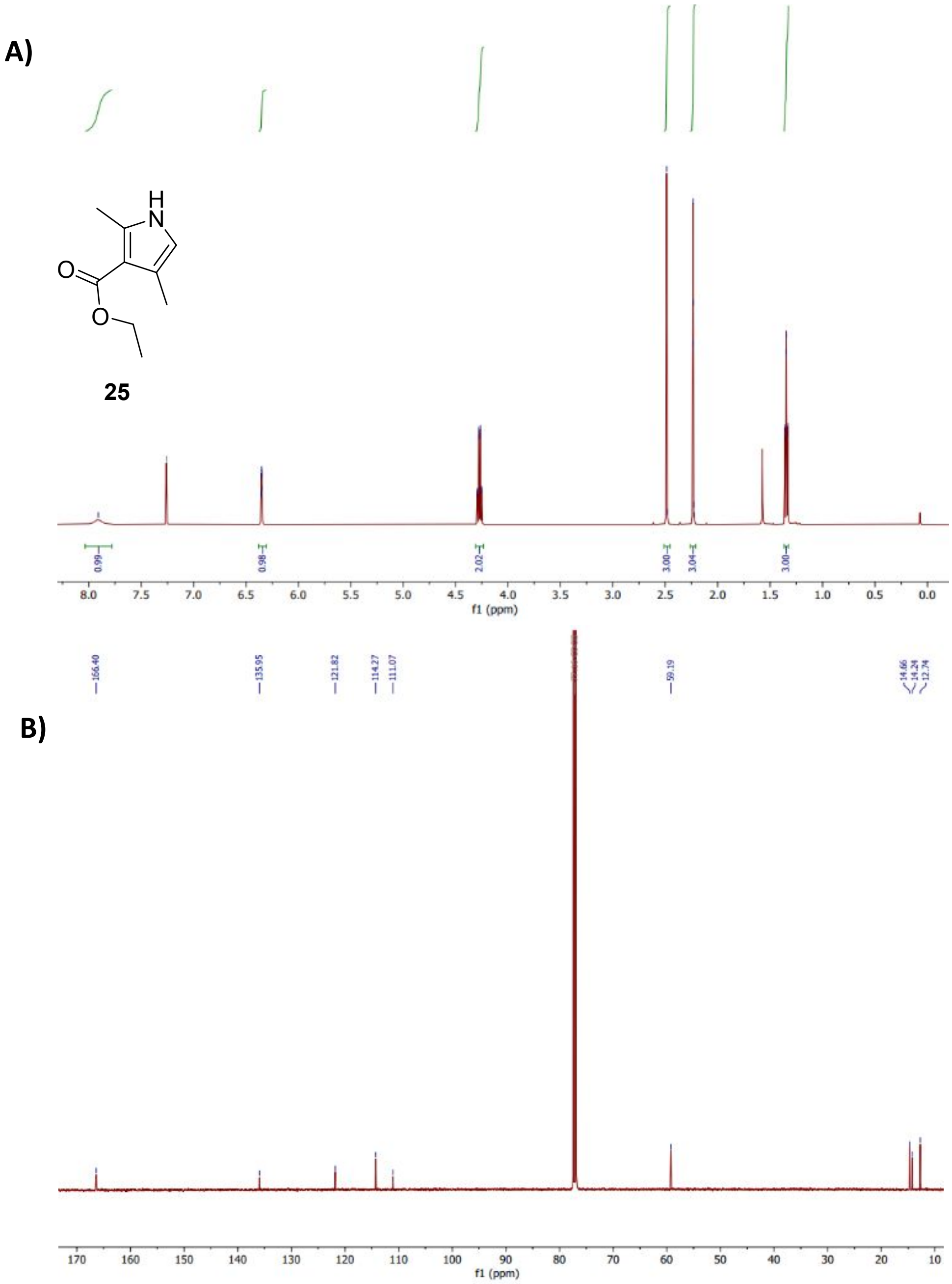


***Figure S16:*** *NMR analysis of pyrrole* ***25*** *purchased from Fluorochem. A)* $^{1}H$ *NMR (500 MHz) and B)* $^{13}C$ *NMR (100 MHz).*

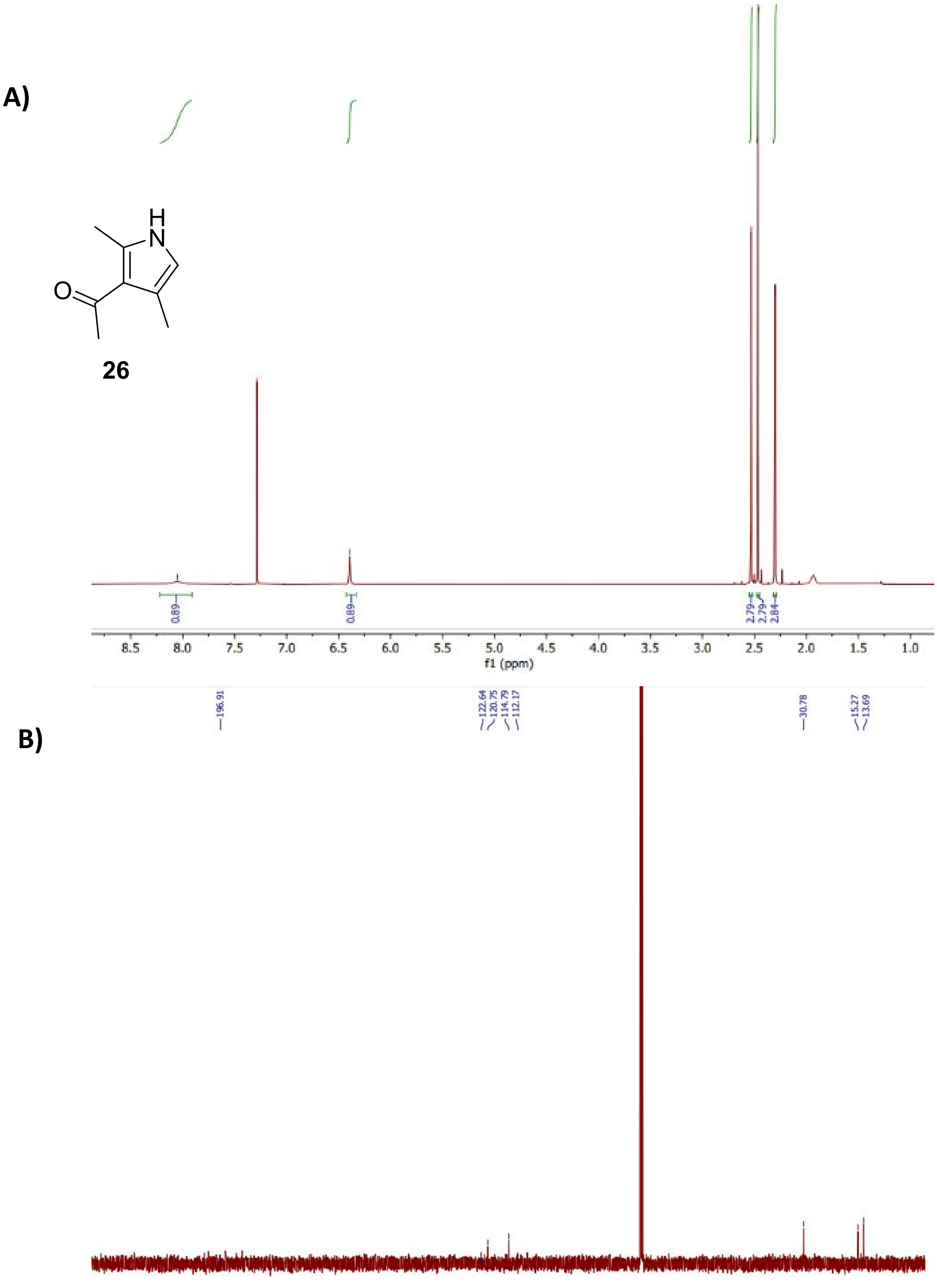


***Figure S17**: NMR analysis of pyrrole **26** isolated from chemical synthesis. A) $^{1}H$ NMR (400 MHz) and B) $^{13}C$ NMR (100 MHz).*

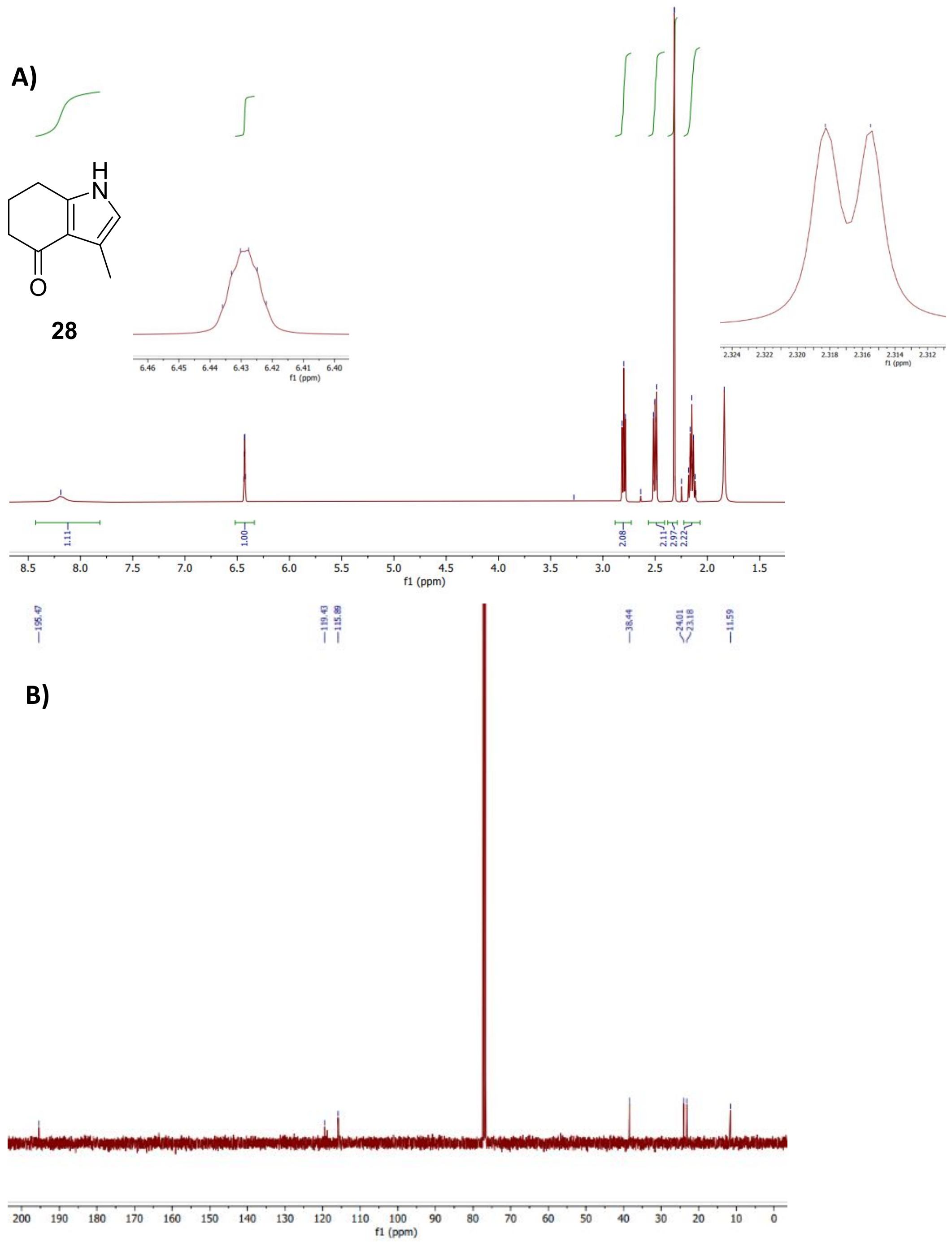


***Figure S18**: NMR analysis of pyrrole **28** isolated from chemical synthesis. A) $^{1}$H NMR (400 MHz). Highlighted is the multiplet at $\sigma$ = 6.42 which is assigned to the pyrrole C-H. The doublet at $\sigma$ = 2.32 is assigned to the pyrrole methyl. B) $^{13}$C NMR (100 MHz.*

# 17. X-Ray Structure of the PLP-Bound Form of *Th*AOS.

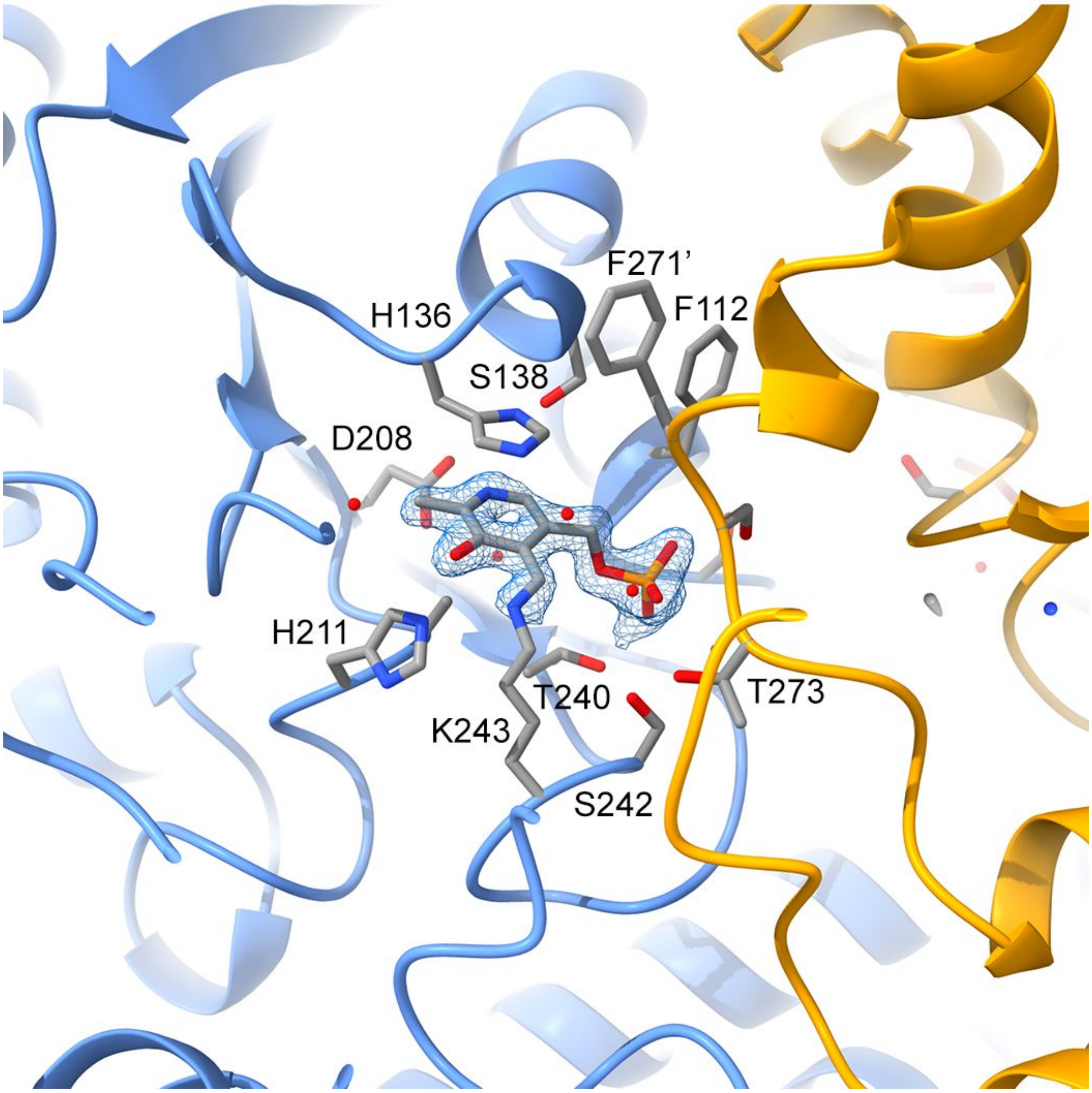


***Figure S19: The ThAOS active site.*** *The crystal structure of* Th*AOS was determined with a PLP-bound in the internal-aldimine resting state with PLP connected to K243 by a Schiff-base linkage. Residues in the vicinity of the PLP are shown in stick representation with the wider protein context shown as cartoons. The PLP and ligand sites sit at the interface of the two monomers, with active-site contributions from both chains. The 2mFo-DFc electron density for the PLP is show at 1.5 sigma. The crystal structure of ThAOS was determined in the internal aldimine, PLP bound, holo-form, featuring the canonical covalent Schiff base between the PLP cofactor and the [illegible]-N atom of the Lys243 side chain. The Re face of the PLP ring is π-stacked against the imidazole sidechain of His136, whilst the N atom of the pyridine PLP ring is chelated by Asp209, suggesting the nitrogen is protonated and positively charged. The conserved phosphate binding cup of AOS enzymes is also present, with the PLP phosphate being bound by Ser110, Thr240, Ser242 and Thr273.*

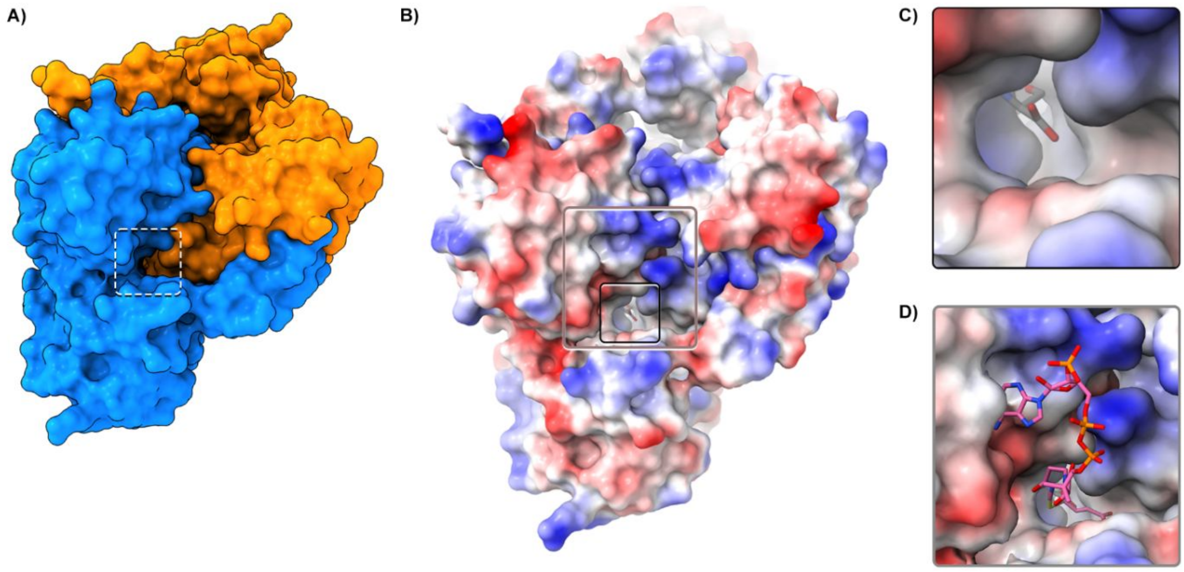


***Figure S20: ThAOS ligand binding tunnel.*** *The ThAOS active site and PLP cofactor are buried in a deep tunnel with a Y-shaped entrance.* ***(A)*** *Surface view showing contribution of both chains in the ThAOS dimer to the active site. Ligand binding region highlighted with a white frame.* ***(B)*** *Electrostatic potential surface of the ThAOS dimer.* ***(C)*** *Inset showing expanded view of entrance to the PLP binding site with docked L-Ser to illustrate accessibility of amino-acid substrates.* ***(D)*** *Proposed position of the acyl-CoA substrate, with modelling based on the co-crystal structure of succinyl-CoA with the aminolevulinic acid synthase (ALAS) from Rhodobacter capsulatus (PDB: 2BWO).*[35]

# 18. Sequence and Structural Alignments

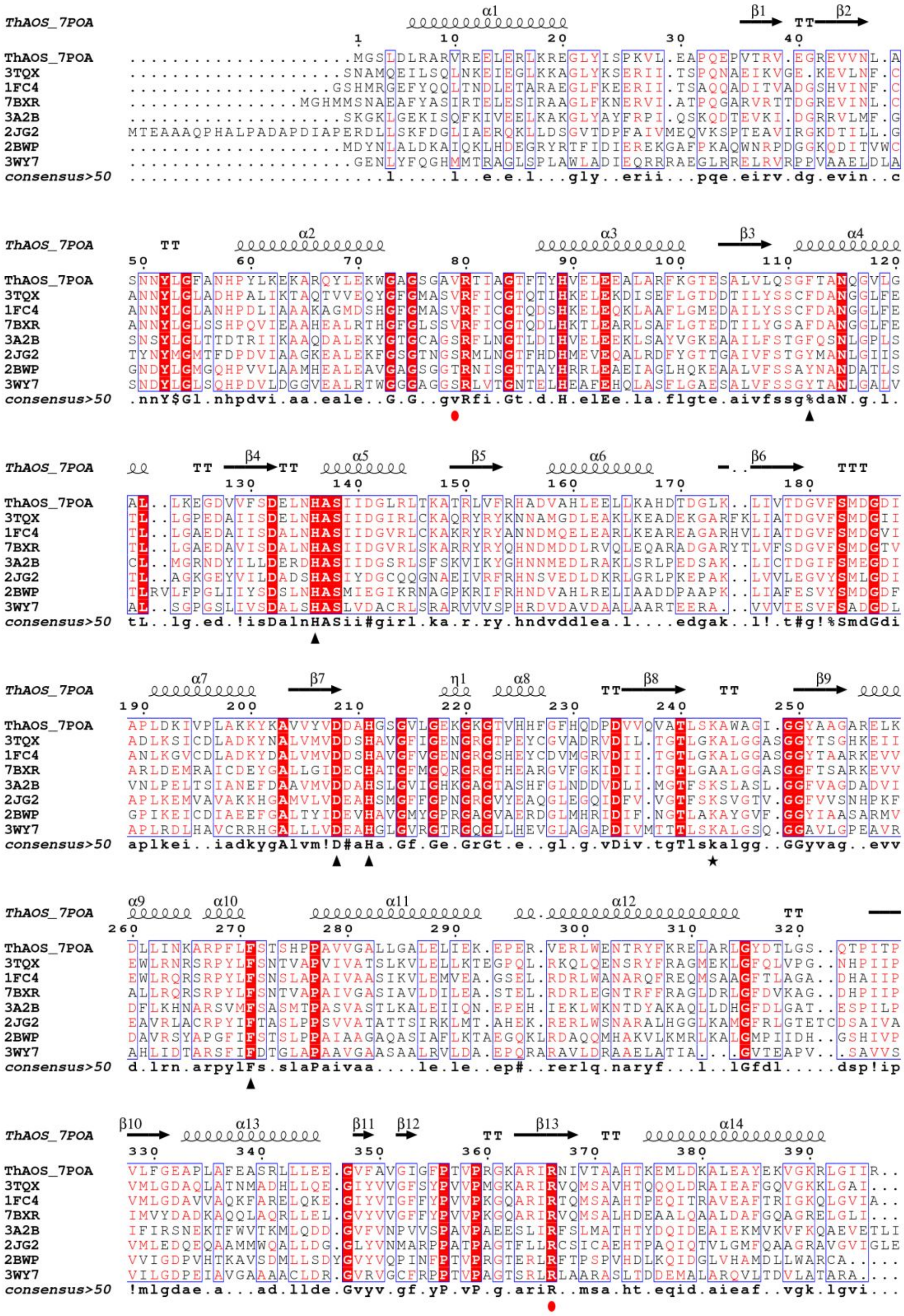

***Figure S21: AOS and related protein family multiple sequence alignment.*** *Annotated multiple sequence alignment of* Th*AOS and some close structural homologues. The sequences of* Th*AOS and homologues from the PDB were aligned using Clustal Omega*[36] *and the alignment was annotated and rendered using ESPript.*[37] *The conserved PLP-binding lysine residue is highlighted with a black star and other conserved PLP-binding residues are highlighted with black triangles; residues which are identified as binding the amino-acid substrates are shown with red ovals. Secondary structure elements from ThAOS (7POA) are shown above the alignment. 3TQX: Coxiella burnetii KBL;*[38] *1FC4: E. coli ketobutyrate ligase (KBL);*[19] *7BXR: Cupriavidus necator KBL (unpublished); 3A2B Sphingobacterium multivorum serine palmitoyltransferase (SPT);*[39] *2JG2: Sphingomonas paucimobilis serine palmitoyltransferase (SPT);*[40] *2BWP: Rhodobacter capsulatus 5-aminolevulinate synthase (ALAS);*[35] *3WY7: Mycobacterium smegmatis 8-amino-7-oxononanoate synthase (AONS).*[41]

# 19. Crystallographic Data

| X-ray data collection and processing statistics* | | | |
|---|---|---|---|
| | *Th*AOS (P1) | *Th*AOS (P2$_1$) | *Th*AOS (P2$_1$2$_1$2$_1$) |
| Beamline | I03 | I03 | I03 |
| Date | 05/05/21 | 05/05/21 | 05/05/21 |
| Wavelength (Å) | 0.9796 | 0.9796 | 0.9796 |
| Resolution (Å) | 55.63-1.60 (1.63-1.60) | 94.64–2.00 (2.03–2.00) | 54.48-2.60 (2.70-2.60) |
| Space group | P1 | P2$_1$ | P2$_1$2$_1$2$_1$ |
| **Unit-cell parameters** | | | |
| a (Å) | 57.41 | 67.10 | 57.04 |
| b (Å) | 63.89 | 57.13 | 134.79 |
| c (Å) | 65.91 | 189.44 | 184.02 |
| α (°) | 64.74 | 90.00 | 90.00 |
| β (°) | 70.50 | 92.35 | 90.00 |
| γ (°) | 68.12 | 90.00 | 90.00 |
| Unit-cell volume (Å$^3$) | 198,438 | 725,667 | 1,414,823 |
| Solvent content (%) | 42 | 53 | 52 |
| No. of measured reflections | 636,458 (26,584) | 1,212,491 (6,322) | 968,949 (99,102) |
| No. of independent reflections | 97,563 (4286) | 97,294 (4797) | 44,495 (4583) |
| Completeness (%) | 96.2 (85.6) | 99.9 (99.3) | 99.7 (100.0) |
| Redundancy | 6.5 (6.2) | 12.5 (13.0) | 21.8 (21.6) |
| CC$_{1/2}$ (%) | 0.997 (0.604) | 0.999 (0.739) | 0.999 (0.775) |
| <I>/<σ(I)> | 15.6 (1.4) | 11.0 (1.5) | 11.6 (1.6) |
| **Model refinement statistics** | | | |
| Rwork (%) | 16.9 | 21.1 | 23.1 |
| Rfree[#] (%) | 23.9 | 26.1 | 31.1 |
| No. of non-H atoms | | | |
| No. of protein, atoms | 6,141 | 12,244 | 12,182 |
| No. of solvent atoms | 488 | 494 | 49 |
| No. of ligand atoms | 51 | 60 | 60 |
| **R.M.S. deviation from ideal values** | | | |
| Bond angle (°) | 1.76 | 1.69 | 2.47 |
| Bond length (Å) | 0.012 | 0.010 | 0.019 |
| **Average B factor (Å$^2$)** | | | |
| Protein | 30 | 38 | 63 |
| Solvent | 38 | 35 | 48 |
| Ligand | 30 | 32 | 48 |
| Ramachandran plot[+], residues in | | | |
| Most favoured regions (%) | 97.46 | 97.65 | 94.69 |
| PDBID | 7POA | 7POB | 7POC |

***Table S5: X-ray data collection and processing statistics****

*(Values in parenthesis are for the highest resolution shell).

[#]5% of the randomly selected reflections excluded from refinement.

[+]Calculated using MOLPROBITY.

# 20. DNA and Protein Sequences

The *Th*AOS gene sequence was codon optimized for *E. coli* expression and prepared as a synthetic gene by GenScript:

ATCGATCTCGATCCCGCGAAATTAATACGACTCACTATAGGGGAATTGTGAGCGGATAACAATTCCCCTCTAG
AAATAATTTTGTTTAACTTTAAGAAGGAGATATACATATGTCGTACTACCATCACCATCACCATCACGATTACG
ACATCCCAACGACCGAAAACCTGTATTTTCAGGGCGCCATGGGCAGCCTGGATCTGCGTGCGCGTGTGCGTG
AAGAACTGGAGCGTCTGAAGCGTGAAGGTCTGTATATTAGCCCGAAAGTGCTGGAAGCGCCGCAGGAACCG
GTGACCCGTGTTGAAGGCCGTGAGGTGGTTAACCTGGCGAGCAACAACTACCTGGGTTTTGCGAACCACCCG
TATCTGAAGGAAAAAGCGCGTCAATACCTGGAGAAATGGGGTGCGGGTAGCGGTGCGGTGCGTACCATCGC
GGGCACCTTCACCTATCACGTTGAACTGGAGGAAGCGCTGGCGCGTTTTAAAGGTACCGAGAGCGCGCTGGT
GCTGCAGAGCGGTTTCACCGCGAACCAAGGCGTTCTGGGTGCGCTGCTGAAGGAAGGCGACGTGGTTTTTAG
CGATGAGCTGAACCACGCGAGCATCATTGACGGTCTGCGTCTGACCAAAGCGACCCGTCTGGTGTTCCGTCAC
GCGGATGTTGCGCACCTGGAGGAACTGCTGAAGGCGCACGACACCGATGGTCTGAAACTGATTGTGACCGAC
GGCGTTTTTAGCATGGACGGTGATATCGCGCCGCTGGATAAGATTGTGCCGCTGGCGAAGAAATACAAAGCG
GTGGTTTATGTGGACGATGCGCACGGCAGCGGTGTTCTGGGCGAAAAGGGCAAAGGTACCGTGCACCACTTC
GGTTTTCACCAGGACCCGGATGTGGTTCAAGTGGCGACCCTGAGCAAAGCGTGGGCGGGTATCGGTGGCTAC
GCGGCGGGTGCGCGTGAGCTGAAGGACCTGCTGATTAACAAAGCGCGTCCGTTCCTGTTTAGCACCAGCCAC
CCGCCGGCGGTGGTTGGTGCGCTGCTGGGTGCGCTGGAACTGATCGAGAAGGAACCGGAGCGTGTGGAACG
TCTGTGGGAGAACACCCGTTATTTCAAACGTGAGCTGGCGCGTCTGGGCTACGATACCCTGGGTAGCCAGACC
CCGATCACCCCGGTTCTGTTCGGTGAAGCGCCGCTGGCGTTTGAGGCGAGCCGTCTGCTGCTGGAGGAAGGC
GTGTTCGCGGTTGGCATTGGTTTTCCGACCGTGCCGCGTGGTAAAGCGCGTATCCGTAACATTGTTACCGCGG
CGCACACCAAAGAGATGCTGGACAAGGCGCTGGAGGCGTATGAAAAGGTTGGCAAACGTCTGGGTATTATCC
GCTAACAAAGCCCGAAAGGAAGCTGAGTTGCTGCTGCCACCGCTGAGCAATAACTAGCATAACCCCTTGGGG
CCTCTAAACGGGTCTTGAGGGGTTTTTTGCTGAA

Expression construct:
MSYYHHHHHHDYDIPTTENLYFQGAMGSLDLRARVREELERLKREGLYISPKVLEAPQEPVTRVEGREVVNLASNN
YLGFANHPYLKEKARQYLEKWGAGSGAVRTIAGTFTYHVELEEALARFKGTESALVLQSGFTANQGVLGALLKEGD
VVFSDELNHASIIDGLRLTKATRLVFRHADVAHLEELLKAHDTDGLKLIVTDGVFSMDGDIAPLDKIVPLAKKYKAVV
YVDDAHGSGVLGEKGKGTVHHFGFHQDPDVVQVATLSKAWAGIGGYAAGARELKDLLINKARPFLFSTSHPPAVV
GALLGALELIEKEPERVERLWENTRYFKRELARLGYDTLGSQTPITPVLFGEAPLAFEASRLLLEEGVFAVGIGFPTVPR
GKARIRNIVTAAHTKEMLDKALEAYEKVGKRLGIIR

The underlined sequence at the N-terminus is removed upon TEV cleavage.

The *Ec*ACS gene sequence (Uniprot: P27550) was cloned from *E. coli* O157 chromosomal DNA into a pET-TEV-cleavable N-$His_6$ expression plasmid:

TGTTGGAGGAAATTCCCCTCTAGAATAATTTTGTTTAACTTTAAGAAGGAGATATACATATGTCGTACTACCAT
CACCATCACCATCACGATTACGATATCCCAACGACCGAAAACCTGTATTTTCAGGGCGCCATGAGCCAAATTCA
CAAACACACCATTCCTGCCAACATCGCAGACCGTTGCCTGATAAACCCTCAGCAGTACGAGGCGATGTATCAA
CAATCTATTAACGTACCTGATACCTTCTGGGGCGAACAGGGAAAAATTCTTGACTGGATCAAACCTTACCAGA

AGGTGAAAAACACCTCCTTTGCCCCCGGTAATGTGTCCATTAAATGGTACGAGGACGGCACGCTGAATCTGGCGGCAAACTGCCTTGACCGCCATCTGCAAGAAAACGGCGATCGTACCGCCATCATCTGGGAAGGCGACGACGCCAGCCAGAGCAAACATATCAGCTATAAAGAGCTGCACCGCGACGTCTGCCGCTTCGCCAATACCCTGCTCGAGCTGGGCATTAAAAAAGGTGATGTGGTGGCGATTTATATGCCGATGGTGCCGGAAGCCGCGGTTGCGATGCTGGCCTGCGCCCGCATTGGCGCGGTGCATTCGGTGATTTTCGGCGGCTTCTCGCCGGAAGCCGTTGCCGGGCGCATTATTGATTCCAACTCACGACTGGTGATCACTTCCGACGAAGGTGTGCGTGCCGGGCGCAGTATTCCGCTGAAGAAAAACGTTGATGACGCGCTGAAAAACCCGAACGTCACCAGCGTAGAGCATGTGGTGGTACTGAAGCGTACTGGCGGGAAAATTGACTGGCAGGAAGGGCGCGACCTGTGGTGGCACGACCTGGTTGAGCAAGCGAGCGATCAGCACCAGGCGGAAGAGATGAACGCCGAAGATCCGCTGTTTATTCTCTACACCTCCGGTTCTACCGGTAAGCCAAAAGGTGTGCTGCATACTACCGGCGGTTATCTGGTGTACGCGGCGCTGACCTTTAAATATGTCTTTGATTATCATCCGGGTGATATCTACTGGTGCACCGCCGATGTGGGCTGGGTGACCGGACACAGTTACTTGCTGTACGGCCCGCTGGCCTGCGGTGCGACCACGCTGATGTTTGAAGGCGTACCCAACTGGCCGACGCCTGCCCGTATGGCGCAGGTGGTGGACAAGCATCAGGTCAATATTCTCTATACCGCACCCACGGCGATCCGCGCGCTGATGGCGGAAGGCGATAAAGCGATCGAAGGCACCGACCGTTCGTCGCTGCGCATTCTCGGTTCCGTGGGCGAGCCAATTAACCCGGAAGCGTGGGAGTGGTACTGGAAAAAAATCGGCAACGAGAAATGTCCGGTGGTCGATACCTGGTGGCAGACCGAAACCGGCGGTTTCATGATCACCCCGCTGCCTGGCGCTACCGAGCTGAAAGCCGGTTCGGCAACACGTCCGTTCTTCGGCGTGCAACCGGCGCTGGTCGATAACGAAGGTAACCCGCTGGAGGGGGCCACCGAAGGTAGCCTGGTAATCACCGACTCCTGGCCGGGTCAGGCGCGTACGCTGTTTGGCGATCACGAACGTTTTGAACAGACCTACTTCTCCACCTTCAAAAATATGTATTTCAGCGGCGACGGCGCGCGTCGCGATGAAGATGGCTATTACTGGATAACCGGGCGTGTGGACGACGTGCTGAACGTCTCCGGTCACCGTCTGGGGACGGCAGAGATTGAGTCGGCGCTGGTGGCGCATCCGAAGATTGCCGAAGCCGCCGTAGTAGGTATTCCGCACAATATTAAAGGTCAGGCGATCTACGCCTACGTCACGCTTAATCACGGGGAGGAACCGTCACCAGAACTGTACGCAGAAGTCCGCAACTGGGTGCGTAAAGAGATTGGCCCGCTGGCGACGCCAGACGTGCTGCACTGGACCGACTCCCTGCCTAAAACCCGCTCCGGCAAAATTATGCGCCGTATTCTGCGCAAAATTGCGGCGGGCGATACCAGCAACCTGGGCGATACCTCGACGCTTGCCGATCCTGGCGTAGTCGAGAAGCTGCTTGAAGAGAAGCAGGCTATCGCGATGCCATCGTAA

Expression construct:

MSYYHHHHHHDYDIPTTENLYFQGAMSQIHKHTIPANIADRCLINPQQYEAMYQQSINVPDTFWGEQGKILDWIKPYQKVKNTSFAPGNVSIKWYEDGTLNLAANCLDRHLQENGDRTAIIWEGDDASQSKHISYKELHRDVCRFANTLLELGIKKGDVVAIYMPMVPEAAVAMLACARIGAVHSVIFGGFSPEAVAGRIIDSNSRLVITSDEGVRAGRSIPLKKNVDDALKNPNVTSVEHVVVLKRTGGKIDWQEGRDLWWHDLVEQASDQHQAEEMNAEDPLFILYTSGSTGKPKGVLHTTGGYLVYAALTFKYVFDYHPGDIYWCTADVGWVTGHSYLLYGPLACGATTLMFEGVPNWPTPARMAQVVDKHQVNILYTAPTAIRALMAEGDKAIEGTDRSSLRILGSVGEPINPEAWEWYWKKIGNEKCPVVDTWWQTETGGFMITPLPGATELKAGSATRPFFGVQPALVDNEGNPLEGATEGSLVITDSWPGQARTLFGDHERFEQTYFSTFKNMYFSGDGARRDEDGYYWITGRVDDVLNVSGHRLGTAEIESALVAHPKIAEAAVVGIPHNIKGQAIYAYVTLNHGEEPSPELYAEVRNWVRKEIGPLATPDVLHWTDSLPKTRSGKIMRRILRKIAAGDTSNLGDTSTLADPGVVEKLLEEKQAIAMPS-

# 21. References


1. Vonrhein, C.; Flensburg, C.; Keller, P.; Sharff, A.; Smart, O.; Paciorek, W.; Womack, T.; Bricogne, G., Data processing and analysis with the autoPROC toolbox. *Acta Crystallogr.* **2011,** *67* (4), 293-302.

2. Winter, G., Xia2: An expert system for macromolecular crystallography data reduction. *J. App. Crystallogr.* **2010,** *43* , 186-190.

3. Karplus, P. A.; Diedrerichs, K., Assessing and maximizing data quality in macromolecular crystallography. *Curr. Op. Struct. Biol.* **2015,** *34*, 60-68.

4. Evans, P., Scaling and assessment of data quality. *Acta Crystallogr. D* **2006,** *62*, 72-82.

5. Evans, P. R., An introduction to data reduction: Space-group determination, scaling and intensity statistics. *Acta Crystallogr. D* **2011,** *67*, 282-292.

6. McCoy, A. J.; Grosse-Kunstleve, R. W.; Adams, P. D.; Winn, M. D.; Storoni, L. S.; Read, R. J., Phaser crystallographic software. *J. App. Crystallogr.* **2007,** *40*, 658-674.

7. Franklin, M. C.; Jonah, C.; Rudolph, M. J.; Burshteyn, F.; Cassidy, M.; Ebony, G.; Brandan, H.; Yao, Z. K.; Carlier, P. R.; Totrov, M.; Love, J. D., Structural genomics for drug design against the pathogen Coxiella burnetii. *Proteins: Struct. Funct. Genet.* **2015,** *83*, 2124-2136.

8. Cowtan, K., The Buccaneer software for automated model building. 1. Tracing protein chains. *Acta Crystallogr. D* **2006,** *62*, 1002-1011.

9. Murshudov, G. N.; Pavol, S.; Lebedev, A. A.; Pannu, N. S.; Steiner, R. A.; Nicholls, R. A.; Winn, M. D.; Long, F.; Vagin, A. A., REFMAC5 for the refinement of macromolecular crystal structures. *Acta Crystallogr. D* **2011,** *67*, 355-367.

10. Emsley, P.; Lohkamp, B.; Scott, W. G.; Cowtan, K., Features and development of Coot. *Acta Crystallogr. D* **2010,** *66*, 486-501.

11. Williams, C. J.; Headd, J. J.; Moriarty, N. W.; Prisant, M. G.; Videau, L. L.; Deis, L. N.; Verma, V.; Keedy, D. A.; Hintze, B. J.; Chen, V. B.; Jain, S.; Lewis, S. M., MolProbity: More and better reference data for improved all-atom structure validation. *Prot. Sci.* **2018,** *27*, 293-315.

12. Krissinel, E. B.; Uski, V.; Lebedev, A. A.; Winn, M. D.; Ballard, C., Distributed computing for macromolecular crystallography. *Acta Crystallogr. D* **2018,** *74*, 143-151.

13. Winn, M. D.; Ballard, C. C.; Cowtan, K. D.; Dodson, E. J.; Emsley, P.; Evans, P. R.; Keegan, R. M.; Krissinel, E. B.; Leslie, A. G. W.; McCoy, A.; McNicholas, S. J.; Murshudov, G. N.; Pannu, N. S.; Potterton, E. A.; Powell, H. R.; Read, R. J.; Vagin, A.; Wilson, K. S., Overview of the CCP4 suite and current developments. *Acta Crystallogr. D* **2011,** *67*, 235-242.

14. Pettersen, E. F.; Goddard, T. D.; Huang, C. C.; Meng, E. C.; Couch, G. S.; Croll, T. I.; Morris, J. H.; Ferrin, T. E., UCSF ChimeraX: Structure visualization for researchers, educators, and developers. *Prot. Sci.* **2021,** *30*, 70-82.

15. Shiraiwa, Y.; Ikushiro, H.; Hayashi, H., Multifunctional Role of His159 in the Catalytic Reaction of Serine Palmitoyltransferase. *J. Biol. Chem.* **2009,** *284*, 15487-15495.

16. Webster, S. P.; Alexeev, D.; Campopiano, D. J.; Watt, R. M.; Alexeeva, M.; Sawyer, L.; Baxter, R. L., Mechanism of 8-amino-7-oxononanoate Synthase: Spectroscopic, Kinetic and Crystallographic Studies. *Biochem.* **2000,** *39*, 516-528.

17. Hunter, G. A.; Ferreira, G. C., Molecular enzymology of 5-aminolevulinate synthase, the gatekeeper of heme biosynthesis. *Biochim. Biophys. Act.* **2011,** *1814*, 1467-1473.

18. Webster, S. P.; Campopiano, D. J.; Alexeev, D.; Alexeeva, M.; Watt, R.; Sawyer, L.; Baxter, R. L., Characterisation of 8-amino-7-oxononanoate synthase: A bacterial PLP-dependent, acyl CoA condensing enzyme. *Biochem. Soc. Trans.* **1998,** *26*, 268.

19. Schmidt, A.; Sivaraman, J.; Li, Y.; Larocque, R.; Barbosa, J. A. R. G.; Smith, C.; Matte, A.; Schrag, J. D.; Cygler, M., Three-Dimensional Structure of 2-amino-3-ketobutyrate CoA Ligase from *Escherichia coli* Complexed with a PLP-Substrate Intermediate: Inferred Reaction Mechanism. *Biochem.* **2001,** *40*, 5151-5160.

20. Ferreira, G. C.; Gong, J., 5-Aminolevulinate synthase and the first step of heme biosynthesis. *J. Bioenerg. Biomembr.* **1995,** *27*, 151-159.

21. Ikushiro, H.; Hayashi, H.; Kagamiyama, H., A Water-soluble Homodimeric Serine Palmitoyltransferase from *Sphingomonas paucimobilis* EY2395$^T$ Strain. *J. Biol. Chem.* **2001,** *276*, 18249-18256.
22. Wei, Y.; Perez, L. J.; Ng, W. L.; Semmelhack, M. F.; Bassler, B. L., Mechanism of *Vibrio cholerae* autoinducer-1 biosynthesis. *ACS Chem. Bio.* **2011,** *6*, 356-365.
23. Chun, S. W.; Hinze, M. E.; Skiba, M. A.; Narayan, A. R. H., Chemistry of a Unique Polyketide-like Synthase. *J. Am. Chem. Soc.* **2018,** *140*, 2430-2433.
24. Marchetti, P. Exploring and exploiting the enzymes involved in tambjamine YP1 natural product biosynthesis. PhD thesis, University of Edinburgh, 2019 (http://hdl.handle.net/1842/35708).
25. Stanley, A. E.; Walton, L. J.; Zerikly, M. K.; Corre, C.; Challis, G. L., Elucidation of the *Streptomyces coelicor* pathway to 4-methoxy-2,2'-bipyrrole-5-carboxaldehyde, an intermediate in prodiginine biosynthesis. *Chem. Comm.* **2006,** 3981-3983.
26. Hu, D. X.; Withall, D. M.; Challis, G. L.; Thomson, R. J., Structure, Chemical Synthesis, and Biosynthesis of Prodiginine Natural Products. *Chem. Rev.* **2016,** *116*, 7818-7853.
27. Gerber, R.; Lou, L.; Du, L., A PLP-dependent polyketide chain releasing mechanism in the biosynthesis of mycotoxin fumonisins in *Fusarium verticilliodes*. *J. Am. Chem. Soc.* **2009,** *131*, 3148-3149.
28. Kawata, J.; Naoe, T.; Ogasawara, Y.; Dairi, T., Biosynthesis of the Carbonylmethylene Structure Found in the Ketomemicin Class of Pseudopeptides. *Angew. Chem. Int.* **2017,** *56*, 2026-2029.
29. Rebets, Y.; Nadmid, S.; Paulus, C.; Dahlem, C.; Herrmann, J.; Hgbner, H.; Rgckert, C.; Kiemer, A. K.; Gmeiner, P.; Kalinowski, J.; Mgller, R.; Luzhetskyy, A., Perquinolines A-C: Unprecedented Bacterial Tetrahydroisoquinolines Involving an Intriguing Biosynthesis. *Angew. Chem. Int. Ed.* **2019,** *58*, 12930-12934.
30. Masuo, S.; Tsuda, Y.; Namai, T.; Minakawa, H.; Shigemoto, R.; Takaya, N., Enzymatic cascade in *Psuedomonas* for pyrazine production from $\alpha$-amino acids. *Chem. Bio. Chem.* **2020,** *21*, 353-359.
31. Dai, G. Z.; Han, W. B.; Mei, Y. N.; Xu, K.; Jiao, R. H.; Ge, H. M.; Tan, R. X., Pyridoxal-5'-phosphate-dependent bifunctional enzyme catalysed biosynthesis of indolizidine alkaloids in fungi. *Proc. Natl. Acad. Sci. U.S.A.* **2020,** *117*, 1174-1180.
32. Zhou, T.; Gao, D.; Li, J. X.; Xu, M. J.; Xu, J., Identification of an $\alpha$-Oxoamine Synthase and a One-Pot Two-Step Enzymatic Synthesis of $\alpha$-Amino Ketones. *Org. Lett.* **2020,** *23*, 37-41.
33. Richardson, S. M.; Harrison, P. J.; Herrera, M. A.; Wang, M.; Verez, R.; Ortiz, G. P.; Campopiano, D. J., BioWF: A Naturally-Fused, Di-Domain Biocatalyst from Biotin Biosynthesis Displays an Unexpectedly Broad Substrate Scope. *ChemBioChem* **2022,** *23*, e202200171.
34. Liaw, C. C.; Lo, L. H.; Cheng, T. H.; Chan, Y. T.; Huang, Y. R.; Wang, A. H. J.; Chang, H. Y., Biosynthesis of Vitroprocines by α-Oxoamine Synthase and Oxidoreductase Identified from Vibrio sp. QWI-06. *Org. Lett.* **2022,** *24*, 3281-3285.
35. Astner, I.; Schulze, J. O.; van der Heuvel, J.; Jahn, D.; Schubert, W. D.; Heinz, D. W., Crystal structure of 5-aminolevulinate synthase, the first enzyme of heme biosynthesis, and its link to XSLA in humans. *EMBO* **2005,** *24*, 3166-3177.
36. Sievers, F.; Wilm, A.; Dineen, D.; Gibson, T. J.; Karplus, K.; Li, W.; Lopez, R.; McWilliam, H.; Remmert, M.; Soding, J.; Thompson, J. D.; Higgins, D. G., Fast, scalable generation of high-quality protein multiple sequence alignments using Clustal Omega. *Mol. Syst. Biol.* **2011,** *7*, 539.
37. Robert, X.; Gouet, P., Deciphering key features in protein structures with the new ENDscript server. *Nucleic Acids Research* **2014,** *42* (W1), W320-W324.
38. Franklin, M. C.; Cheung, J.; Rudolph, M. J.; Burhsteyn, F.; Cassidy, M.; Gary, E.; Hillerich, B.; Yao, Z. K.; Carlier, P. R.; Totrov, M.; Love, J. D., Structural genomics for drug design against the pathogen *Coxiella burnetii*. *Proteins* **2015,** *83*, 2124-2136.
39. Ikushiro, H.; Islam, M. M.; Okamoto, A.; Hayashi, H., Structural insights into the enzymatic mechanism of serine palmitoyltransferase from Sphingobacterium multivorum. *J. Biochem.* **2009,** *146*, 549-562.

40. Yard, B. A.; Carter, L. G.; Johnson, K. A.; Overton, I. M.; Dorward, M.; Liu, H.; McMahon, S. A.; Oke, M.; Puech, D.; Barton, G. J.; Naismith, J. H.; Campopiano, D. J., The Structure of Serine Palmitoyltransferase; Gateway to Sphingolipid Biosynthesis. *J. Mol. Biol.* **2007,** *370*, 870-886.
41. Fan, S.; Li, D. F.; Wang, D. C.; Fleming, J.; Zhang, H.; Zhou, Y.; Zhou, L.; Zhou, J.; Chen, T.; Chen, G.; Zhang, X. E.; Bi, L., Structure and function of *Mycobacterium smegmatis* 7-keto-8-aminopelargonic acid (KAPA) synthase. *Int. Journ. Biochem. & Cell Biol.* **2015,** *58*, 71-80.
